# Development and validation of SEER (Seeking, Engaging with and Evaluating Research): a measure of policymakers’ capacity to engage with and use research

**DOI:** 10.1186/s12961-016-0162-8

**Published:** 2017-01-17

**Authors:** Sue E. Brennan, Joanne E. McKenzie, Tari Turner, Sally Redman, Steve Makkar, Anna Williamson, Abby Haynes, Sally E. Green

**Affiliations:** 1 School of Public Health and Preventive Medicine, Monash University, Melbourne, Australia; 2 Sax Institute, Sydney, Australia; 3 School of Public Health, University of Sydney, Sydney, Australia

**Keywords:** Evidence-informed policy, Research utilisation, Knowledge translation, Knowledge exchange, Capacity to use research, Capacity building, Measurement instrument, Questionnaire, Conceptual framework, Health policy

## Abstract

**Background:**

Capacity building strategies are widely used to increase the use of research in policy development. However, a lack of well-validated measures for policy contexts has hampered efforts to identify priorities for capacity building and to evaluate the impact of strategies. We aimed to address this gap by developing SEER (Seeking, Engaging with and Evaluating Research), a self-report measure of individual policymakers’ capacity to engage with and use research.

**Methods:**

We used the SPIRIT Action Framework to identify pertinent domains and guide development of items for measuring each domain. Scales covered (1) individual capacity to use research (confidence in using research, value placed on research, individual perceptions of the value their organisation places on research, supporting tools and systems), (2) actions taken to engage with research and researchers, and (3) use of research to inform policy (extent and type of research use). A sample of policymakers engaged in health policy development provided data to examine scale reliability (internal consistency, test-retest) and validity (relation to measures of similar concepts, relation to a measure of intention to use research, internal structure of the individual capacity scales).

**Results:**

Response rates were 55% (150/272 people, 12 agencies) for the validity and internal consistency analyses, and 54% (57/105 people, 9 agencies) for test-retest reliability. The individual capacity scales demonstrated adequate internal consistency reliability (alpha coefficients > 0.7, all four scales) and test-retest reliability (intra-class correlation coefficients > 0.7 for three scales and 0.59 for fourth scale). Scores on individual capacity scales converged as predicted with measures of similar concepts (moderate correlations of > 0.4), and confirmatory factor analysis provided evidence that the scales measured related but distinct concepts. Items in each of these four scales related as predicted to concepts in the measurement model derived from the SPIRIT Action Framework. Evidence about the reliability and validity of the research engagement actions and research use scales was equivocal.

**Conclusions:**

Initial testing of SEER suggests that the four individual capacity scales may be used in policy settings to examine current capacity and identify areas for capacity building. The relation between capacity, research engagement actions and research use requires further investigation.

**Electronic supplementary material:**

The online version of this article (doi:10.1186/s12961-016-0162-8) contains supplementary material, which is available to authorized users.

## Background

Capacity to use research is among the factors most commonly targeted by efforts to strengthen the use of research in health policy [1–3]. Widely used capacity building strategies include training for policymakers in finding and interpreting research [4–6], the provision of research resources such as databases and evidence-briefs tailored to policy needs [7–10], and partnerships between policymakers and researchers to co-produce research [11–14]. Investment in these strategies stems from a commitment in both policy and research sectors to capitalise on untapped potential for research to inform policy [15]. Yet, the evidence base required to identify priority areas for capacity building and select high impact strategies is lacking. Very few studies have evaluated whether commonly used strategies achieve important outcomes [16–20] nor is there good evidence about which aspects of capacity most influence the use of research (and, hence, are priorities for capacity building) and which are amenable to change [21–23]. As a result of these evidence gaps, agencies risk investing in strategies ill-matched to their needs while forgoing opportunities to enhance their use of research.

Despite growing recognition of the need to understand capacity for research use in policy agencies, few tools are available to assess capacity in this context [2, 24, 25]. Valid measures are needed to assess current capacity, tailor capacity-building strategies to meet needs, and evaluate the impact of the resulting strategies. Ideally, these measures will be suitable for use in research and by policy agencies seeking to understand and foster their own capacity to use research. Such measures should build on existing knowledge (empirical research and theory), and enable feasible, valid and reliable measurement of the concepts they are designed to measure in the intended context [26, 27]. Tools designed for different levels of measurement (organisational, policy, individual) are needed to match the range of strategies employed; from organisation-wide efforts aiming to develop a receptive climate for using research through to individually-targeted professional development. Measures developed to date have focussed on organisational-level capacity [2, 24, 25], with only one instrument identified for individual-level measurement, and this focused on intention to use research rather than capacity [28] (see Additional file 1 for our analysis of these measures). While there are measures designed for clinical contexts (as reviewed by Squires et al. [29], and more recent examples [30]), the content of these is tailored for health professionals and the measures have not been validated with policymakers. The study reported in the current paper aims to address this gap, through development of SEER (Seeking, Engaging with and Evaluating Research), a self-report measure of individual capacity to engage with and use research.

Individual capacity for using research is a multidimensional concept with little consistency in how it is defined or measured. Capacity is a term widely used in the international development sector, where it is conceived as a multi-level concept (individual, organisational, enabling environment) encompassing four elements: (1) tools, (2) skills, (3) staff and infrastructure, and (4) structures, systems and roles [1]. In the healthcare literature, capacity is increasingly used in relation to building competencies required to implement evidence-based practice (see for example [31, 32]). A systematic review of 145 studies of barriers and facilitators of the use of evidence by policymakers points to key dimensions of capacity pertinent to policy contexts, finding that collaboration, relationships and contact with researchers are the factors most commonly reported as influencing research use [21]. Other attributes of capacity, such as knowledge and skills, have been reported by policymakers to enable their research use [21, 33]. There is, however, little evidence examining the association between these reported factors and the use of research. Policymakers’ knowledge and skills have been shown to predict their use of research [34]; so too have perceptions of the relevance of research, access to databases and professional development, and interaction with researchers [21, 23, 35]. This evidence derives from a small number of studies, using study-specific measures, and no single factor stands out as a strong predictor of research use [21, 23]. While these studies suggest factors pertinent to measuring individual capacity, a coherent framework is needed for measurement.

### Conceptual framework underpinning SEER: the SPIRIT action framework

**Fig. 1:**
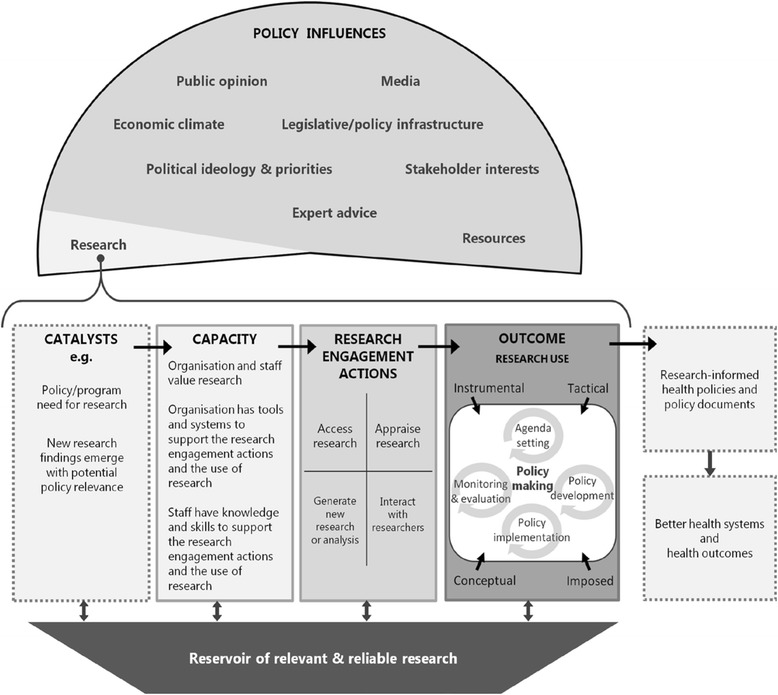
The SPIRIT action framework

The SPIRIT (Supporting Policy In health with Research: an Intervention Trial) Action Framework (Fig. 1) was developed by members of our team to provide greater clarity around the concepts and factors that should be considered when developing and testing interventions intended to support policy agencies in their use of research [36]. The framework specifies potential determinants of research use (individual and organisational capacity), the actions taken to engage with research and researchers (research engagement actions), and the extent to which research actually informs a policy or programme (research use). Co-developed by researchers, policymakers and knowledge exchange specialists, the framework derives from a synthesis of published models and research, insights gained from semi-structured interviews with policymakers, and consultation with experts in the utilisation of research in health policy (detailed analysis reported elsewhere [36]). The SPIRIT Action Framework is undergoing initial testing in a trial examining the effects of a multi-faceted intervention designed to build capacity for research use in policy agencies (the SPIRIT study) [37]. SEER is one of three instruments developed for measuring outcomes of the trial; the others being a self-report measure of organisational capacity (ORACLe) [25] and a direct measure of research engagement and use which is based on interviews and document analysis (SAGE) [38].

#### Domains of the SPIRIT action framework measured by SEER

**Fig. 2:**
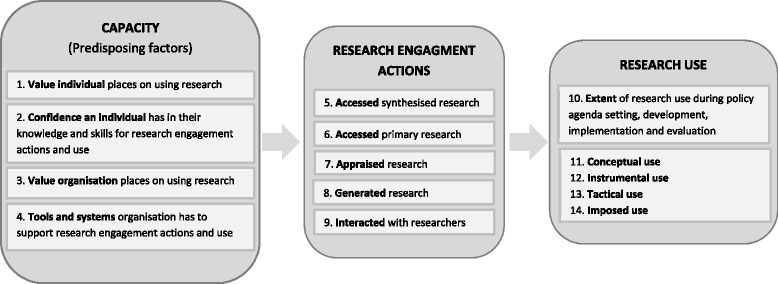
Domains and factors of the SPIRIT Action Framework measured by SEER

Figure 2 shows the domains and factors operationalised in SEER (with factor numbering used through the paper). Although our primary aim was to develop a measure of individual capacity to engage with and use research, SEER includes scales measuring research engagement actions and research use. These are proxy (or indirect) measures of behaviour intended as pragmatic indicators for examining whether individual capacity predicts research engagement and use outcomes. While objective or direct measures of research use, such as derived from SAGE [37], are generally considered to be more accurate (a ‘gold standard’) they are not always feasible [39]. The three domains measured by SEER are described below.

##### Individual capacity

Encompasses factors thought to enable or predispose an individual policymaker to engage with and use research. The SPIRIT Action Framework identifies four predisposing factors, namely (1) the value an individual places on using research, (2) the confidence an individual has in their knowledge and skills for engaging with research, and the perceptions an individual has of (3) the value their organisation places on using research and (4) the tools and systems their organisation has to support research use. Collectively, these factors aim to capture whether an individual has the motivation and capability to engage with research and researchers (research engagement actions). These factors are, therefore, expected to predict research engagement and use. They are also potentially modifiable, so are frequent targets of interventions designed to build capacity for using research. Measuring these factors provides data needed to identify priority areas for intervention and evaluate the effects of capacity building interventions.

##### Research engagement actions

Research engagement actions capture the process of accessing, generating and interpreting research. Four types of research engagement actions are specified in SPIRIT: (5 and 6) accessing synthesised and primary research, (7) appraising research for relevance and quality, (8) generating or commissioning research and analyses, and (9) interacting with researchers. These factors reflect hallmarks of a systematic process for engaging with research (e.g. accessing pre-appraised research) [2]. The actions encompass likely precursors of using research (e.g. accessing research, assessing its relevance) [24] and behaviours thought to pre-dispose policymakers to using research (e.g. collaborating with researchers) [11, 33, 40].

##### Research use

Captures the extent and way in which research is used to inform different stages of policy or programme development. Factors within this domain cover (10) the extent of research use during policy agenda setting and scoping, development, implementation, and evaluation; and whether research is used to (11) understand an issue (conceptual use), (12) develop policy content (instrumental use), (13) persuade (tactical use), or (14) meet organisational requirements (imposed use). Research use is conceived of in the SPIRIT Action Framework as an outcome of research engagement actions moderated by the many contextual factors that influence policy (e.g. resources, political priorities) [36].

### Aims

In this paper, we report on the development and initial testing of SEER, a self-report measure of capacity for engaging with and using research in policy and programme development. SEER measures the perceptions of individual policymakers focussing on the value they place on using research, their confidence in their knowledge and skills to use research, and the extent to which their organisation supports the use of research.

The objectives of the study reported in this paper were (1) to develop a comprehensive self-report measure of individual capacity to engage with and use research, and (2) to assess the properties of the new measure, providing initial evidence about its validity and reliability.

## Methods

Ethics approval for this work was sought and granted from the University of Western Sydney (H9413 11/020863) and Monash University (HREC 2012000062).

We used the Standards for Educational and Psychological Testing to structure reporting of our initial tests of SEER [26]. The COSMIN (COnsensus-based Standards for the selection of health status Measurement Instruments) checklist guided the detailed reporting of methods [41]. The Joint Committee Standards require evidence to support each intended purpose (or interpretation) of a measure. Multiple purposes are proposed for SEER: (1) to describe capacity for engaging with and using research (descriptive); (2) to predict engagement with research and research use (predictive); (3) to discriminate between groups likely to benefit from capacity building interventions and those unlikely to benefit (diagnostic); (4) to measure change in capacity to engage with and use research following exposure to capacity building interventions (evaluative). Our initial tests focus on the first two interpretations, falling mainly within three domains of the Joint Committee Standards: content-related evidence, relations to other variables, and reliability/precision. A test of the internal structure of SEER is also reported for the capacity scales. We define these types of evidence and the testing undertaken for SEER in the methods that follow.

We begin by describing the process used to generate and refine items for SEER. This content-related evidence demonstrates the extent to which the wording and format of items clearly and comprehensively cover the concepts SEER is intended to measure [26].

### Development of items for each domain

The development of items for SEER was informed by analysis of existing instruments that addressed domains of the SPIRIT Action Framework, consultation with researchers with expertise in evidence-informed policy, and multiple rounds of feedback from the broader investigator team contributing to the SPIRIT Action Framework [36]. This investigator team included researchers, policymakers and knowledge exchange specialists. They had subject matter expertise in research utilisation, health policy, evidence-based practice, social research methods, and the development and evaluation of organisational measures.

#### Item generation

Where possible, items were derived or adapted from existing instruments. New items were written by members of the investigator team (TT, SR) for domains for which no suitable items were identified. Item content was based on the operational definitions developed for the SPIRIT Action Framework [36].

#### Item refinement

The items comprising each scale were independently reviewed by others involved in the measures development (AH, SB, SG, JM), then by the broader investigator team contributing to SPIRIT. Development of the SPIRIT Action Framework and SEER occurred in parallel, so those evaluating the content of SEER had working knowledge of the domains and concepts to be measured. Team members evaluated items based on relevance to the intended content domain (i.e. whether individual items appeared to measure the construct intended; whether the items comprising each scale appeared to be a comprehensive measure of the construct), clarity of wording (i.e. whether items were easy to understand, item length), and singularity (i.e. whether each item appeared to measure a single aspect of the intended concept). Feedback was also sought on the acceptability of the measure to policymakers (overall length, appropriate wording), the recall period for items measuring behaviour, the scaling of items, and the clarity of instructions. Responses were collated following each round of feedback; revisions were drafted (TT, SR) and discussed by the measures development team (AH, SB, SG, JM). Major revisions were circulated to the broader investigator team for feedback and agreement.

### Pilot testing with policymakers: feasibility and acceptability

Draft versions of SEER were administered to policymakers in two rounds of pilot testing. Sampling was purposive, with participants drawn from four policy agencies that had existing links with the SPIRIT team. These were trusted informants, chosen based on the potential relevance of SEER to their role and that of their agency, and their willingness to provide critical feedback on the instruments. Eight policymakers completed the first test, data from which were used to examine the feasibility of administering SEER and acceptability to respondents. Pilot testing was done concurrently with that for other SPIRIT measures, both of which involved interviews. The interviews captured policymakers’ views on the appropriateness of the SPIRIT domains, the wording used in measures, and the definitions of research and policymaking included in the instructions for each measure [42].

Major revisions arising from the development and first round of pilot testing included collapsing items to reduce redundancy and respondent burden, re-categorising items and splitting scales to ensure each scale focused on a unitary concept, and rewording items to delineate attitudinal and behavioural items. Following these revisions, 18 policymakers from two agencies completed the second pilot test. During this testing, and initial administration in the SPIRIT trial, we monitored survey completion time and sought feedback on reasons for non-response. SEER was subsequently shortened to reduce respondent burden and increase response rate. The resulting, shorter, version of SEER was used for the psychometric testing.

SEER was administered as an online survey during pilots and testing. This facilitated the use of skip functions (to enable selected scales to be administered only to the subset of respondents to whom they were relevant), while also supporting efficient administration, ease of completion, and reduced risk of data handling errors.

### Testing the measurement properties of SEER

#### Eligibility criteria and sampling frame

Government and not-for-profit organisations in Australia were eligible to participate if a significant proportion of their work was in health policy or programme development. Organisations that were not eligible were (1) those that had participated in pilot testing of SEER, and (2) those enrolled in the SPIRIT study. Sampling of organisations was purposive, aiming to encompass agencies involved in a broad cross section of policy and programme development.

Staff within agencies were eligible to participate if they (1) were employed by the agency (contractors were ineligible), and they (2) drafted, wrote or contributed to health-related policy documents, or (3) developed or contributed to the development of health programmes, or (4) made or contributed significantly to policy decisions about health services, programmes or resourcing.

#### Recruitment and consent of agencies and agency staff

The Chief Executive or a senior manager in each agency was approached by a lead investigator (SG or SR) to gauge their interest in participation. Agencies that expressed interest received a formal invitation and information package describing background to the study and the investigator team, the anticipated time-commitment for staff, the purpose of SEER and procedures for testing, consent procedures, and contact details for further information. Agencies and their staff were advised of the steps taken to protect the anonymity of both agencies and individuals. In return for their time, agencies were offered a 1 ½ hour training workshop chosen from a selection of sessions on using and generating research for policy. Agencies with more than ten respondents were also offered a facilitated discussion of their agency’s aggregate SEER data led by an investigator (SG or SR). The threshold of ten respondents was set to protect individual anonymity.

Agencies that consented to participate were asked to nominate a liaison person to assist with staff recruitment. The liaison provided the investigator team with email addresses for eligible staff. Agencies were asked to ensure staff could opt out prior to their inclusion on the email list. Those on the mailing list were emailed an invitation to participate that included the information package, consent procedures, and a unique link to the online survey with a participant ID and instructions for completion. Individuals were asked to consent by email, and then confirm their consent on the first screen of the online survey.

#### Survey administration and sampling for validity and test-retest reliability analyses

The first administration of the survey contained SEER (Additional file 2) plus a 15-item measure of intention to use research based on the theory of planned behaviour (TPB measure) [28] (see Additional file 3 for description of this measure and items). The TPB measure was administered to examine the relation between SEER scores and scores on scales measuring variables to which SEER was expected to relate.

Additional survey items asked about role and organisational tenure, training (e.g. in the use of research), and the proportion of time spent on different types of policy work (e.g. development, implementation, evaluation). Participants yet to complete the survey received up to two email reminders. Agencies were told how many (but not which) staff had completed, prompting some agencies to send additional reminders to increase their response rate.

Those completing the first survey were emailed an invitation to complete SEER a second time, providing data for test-retest reliability analyses. Invitations were sent 3–4 weeks after initial completion, a period likely to be sufficient to prevent recall but in which no changes in the underlying concepts (e.g. knowledge and skills) were expected. SEER was administered in the same on-line format at each administration, and participants were not given information about their first-round responses or scores [43].

#### Data management and scoring

Response data were automatically collected and coded in a database on a secure server. Variables for each of the concepts measured by SEER were calculated according to a data dictionary for scoring SEER.

#### Interpretability: assessment of missing data and distribution of scores

Analysis of missing responses was performed to identify (1) items frequently missing from otherwise complete scales (potentially indicating that items were difficult to interpret or inappropriately worded), and (2) responses to entire scales missing toward the end of the survey (potentially indicating unacceptable respondent burden) [27, 43]. We also examined the empirical distribution of scores for each item to determine the potential for floor and ceiling effects [27, 43]. Means, standard deviations, and percentiles were calculated and presented graphically using boxplots. Frequencies and percentages of responses to binary and ordinal items and scales were calculated and presented graphically in bar charts.

#### Reliability testing: stability over time (test-retest analysis) and internal consistency

Data from the two administrations of SEER were used to examine the stability of SEER scores over a time period in which no change to the underlying constructs was expected (test-retest reliability). To assess the reliability of the SEER scales, we calculated Cohen’s weighted kappa statistics for categorical scales and intra-class correlations for continuous scales. We used quadratic weights in the calculation of the kappa statistic to weigh the importance of disagreements. This quadratic scheme is recommended in the absence of a rationale for a particular weighting scheme [27]. For variables with three or more values, we calculated confidence intervals for the weighted kappa statistic using bootstrapping. Bias corrected 95% confidence intervals were calculated from 1000 replicates. Kappa values of 1 indicate perfect agreement, 0 indicates agreement equal to that expected by chance, and negative values indicate agreement worse than chance [44]. Kappa’s below 0.6 are typically rated as poor to fair according to commonly applied thresholds [27].

Intra-class correlation coefficients (ICCs) were estimated from multilevel linear regressions with two random effects, namely, agency and participant. The estimated variance components from these models were used to calculate an ICC at the organisation level ($\mathrm{I}\mathrm{C}\mathrm{C}-\mathrm{organisation}=\frac{{\widehat{\sigma}}_{org}^2}{{\widehat{\sigma}}_{org}^2+{\widehat{\sigma}}_{ind}^2+{\widehat{\sigma}}_{residual}^2}$) and an ICC at the individual-within-organisation level ($\mathrm{I}\mathrm{C}\mathrm{C}-\mathrm{test}-\mathrm{retest}=\frac{{\widehat{\sigma}}_{org}^2+{\widehat{\sigma}}_{ind}^2}{{\widehat{\sigma}}_{org}^2+{\widehat{\sigma}}_{ind}^2+{\widehat{\sigma}}_{residual}^2}$). The ICC at the organisation level yields an estimate of correlation between the SEER scores from individuals within the same organisation, thus enabling us to test our prediction that the scale measures an organisational level construct, while the ICC at the individual-within-organisation level yields an estimate of correlation between the SEER scores within the same individual and organisation, thus providing us with an estimate of the test-retest reliability. ICC coefficients range from 0 to 1, with 0 indicating no reliability and 1 perfect reliability (or no measurement error) [27, 45]. For newly developed scales used in research, a commonly reported threshold of acceptable reliability is an ICC coefficient greater than 0.7 [27, 46].

Cronbach’s alpha coefficients were calculated using data from the first administration to assess the internal consistency (factor reliability) of the capacity scales.

#### Relations to other variables: convergent and criterion validity

Data collected from the first administration of SEER enabled testing of whether SEER capacity scores (1) converge with scores on TPB scales measuring similar concepts (convergent validity), and (2) correlated with scores on the TPB scale measuring behavioural intention to use research, an outcome that capacity is expected to predict (criterion or predictive validity). The TPB scales that are most similar to SEER scales are those measuring attitudes toward research (TPB ‘attitudes’ scale, similar to SEER ‘value of research’ scale), self-efficacy (efficacy items from the TPB ‘behavioural control’ scale, similar to SEER ‘confidence’ scale), and social norms (TPB ‘social norms’ scale, similar to SEER ‘organisational value’ scale) (see Additional file 1 for comparison of scales). We specified a priori our hypotheses about the direction and magnitude of correlation for each of the tests of convergence (reported with the results). We calculated correlation coefficients to describe the relationship between the scales using Pearson’s product moment correlation coefficient for continuous scales, the point-biserial correlation coefficient for binary and continuous scales, and Spearman’s rank correlation coefficient for ordinal scales. Confidence intervals for the correlations were calculated using bootstrapping, allowing for clustering of observations within organisation. Bias corrected 95% confidence intervals were calculated from 5000 replicates. The relationship between scales was depicted graphically using scatter plots and box plots.

#### Internal structure

Evidence supporting the internal structure of a measure demonstrates that items within a scale relate as predicted by the measurement model, and can, therefore, be summed to yield a meaningful measure. We used confirmatory factor analysis (CFA) to assess the internal structure of our proposed model for the SEER capacity scales. We first fitted models for each factor separately, and then fitted a full model that allowed for correlation among the latent constructs. The former separate models were fitted to allow examination of how the coefficients changed when the full model was fitted, but our focus is on the results from the full model. We examined modification indices to guide potential changes to the full model. We assessed model fit using the standardised root mean squared residuals (SRMSR) index and the coefficient of determination. The method of maximum likelihood was used to obtain parameter estimates with robust variance-covariance estimation (sandwich variance estimator), to account for the correlation of responses within organisation. Robust maximum likelihood adjusts for non-normality arising from the categorical nature of the variables. The method of robust maximum likelihood has been shown to perform well in circumstances where the ordinal variables have five or more categories and the sample size is small [47]. All of the capacity scales used 5-point Likert scales, except those items measuring the tools and systems an organisation has to support research use, which used 3-point scales.

#### Sample size

We aimed to recruit at least 160 participants to complete the SEER instrument so as to have sufficient accuracy in estimating the correlation coefficients between continuous variables. Specifically, a sample of 160 ensures (assuming bivariate normality) that the difference between the sample estimate and an assumed population correlation of 0.6 (or greater), will be no larger than ± 0.1 approximately 95% of the time [48]. For the reliability testing, we aimed to recruit 55 participants to undertake the retest so as to estimate the ICC with a 95% confidence interval of width of 0.2. This sample size calculation assumed an ICC of 0.8 [49].

## Results

### Scales and items developed for each domain

The items comprising each of the SEER scales, their source, response options and scoring are summarised in Table 1 and listed in Additional file 2. In all cases, a higher score is interpreted as a more desirable perception or action. Scales measuring research engagement actions (scales 5–9) and research use (scales 10–14), were administered only to respondents that indicated they had contributed to development of a policy or programme in the last 6 months.

**Table 1:** Summary of SEER scales, items developed for each domain, and scoring

Domains and factors measured by SEER scales	What the scale measures	Source of items	No. of items	Response options and scoring
*Capacity – predisposing factors*				
1. Value individual places on using research	Individual policymakers’ views on the value of research for informing each stage of policy work (e.g. deciding on policy content, designing evaluation)	New items were written for this scale because no suitable scales or items were identified	7	Five-point adjectival scale ranging from “not at all valuable” (score = 1) to “very valuable” (score = 5); scores are summed across items to create a scale score (range 7 to 35)
2. Confidence in using research	Individual policymakers’ confidence in their ability (knowledge and skills) to engage with research (by accessing, appraising, generating and applying research) and researchers; items from these instruments were not suitable for measuring individual knowledge or skills	New items were written for this scale, informed by the concepts covered in two measures of organisational capacity (‘Is research working for you?’ [24, 55] and SUPPORT [2]; for analysis, see Additional file 1)	7	Five-point adjectival scale ranging from “not at all confident” (score = 1) to “very confident” (score = 5); scores are summed across items to create a scale score (range 7 to 35)
3. Value organisation places on research use	Individual policymakers’ perceptions of leaders’ beliefs and organisational expectations about the use of research	New items were written for this scale, informed by the concepts measured by the Canadian Institutes of Health Research (CIHR) and SUPPORT instruments	5	Five-point adjectival scale ranging from “never” (score = 1) to “always” (score = 5); scores are summed across items to create a scale score (range 5 to 25)
4. Tools and systems organisation has to support research use	Individual policymakers’ perceptions of the supports their organisation has in place for training, accessing research, guiding policy evaluation and research commissioning, and engaging with researchers	New items were written for this scale, informed by the CIHR and SUPPORT instruments	7	Four response options: ‘no’ (organisation does not have this tool or system) (score = 1), ‘yes, but limited’ (score = 2), ‘yes, well developed’ (score = 3), or ‘I don’t know’ (recoded as ‘no’, reflecting the interpretation that lack of awareness of support suggests a support that is not functional) Scores are summed across items to create a scale score (range 7 to 21)
*Research engagement actions*				
5. Accessed synthesised research	Whether individual policymakers searched for or commissioned reviews of research over the last 6 months; responses were in relation to the policy on which most time had been spent	New items were written for this scale	2	Binary response to individual items (yes/no) A ‘yes’ response to either or both items attracts the maximum score doing both actions (commissioning or searching for syntheses) is unlikely to be necessary
6. Accessed primary research	Whether individual policymakers searched for single studies or government websites over the last 6 months; responses were in relation to the policy on which most time had been spent	New items were written for this scale	2	Binary response to individual items (yes/no) Items are summed to create a scale score (ordinal scale score: 0, 1, 2)
7. Appraised research	Whether individual policymakers assessed the methods, reliability of results, and generalisability of research used to inform a specific policy over the last 6 months; responses were in relation to the policy on which most time had been spent	New items were written for this scale	3	Binary response to individual items (yes/no) Items are summed to create a scale score (ordinal scale score: 0, 1, 2, 3) Items are administered only if respondents answer ‘yes’ to an item asking if they found research
8. Generated research	Whether individual policymakers generated research or analyses to inform a specific policy through an internally conducted project, commissioning or partnering with researchers, or evaluation of a policy or program; responses were in relation to the last 6 months and the policy on which most time had been spent	One item was adapted from Campbell et al.’s [11] five item scale measuring links with researchers and two new items were written	3	Binary response to individual items (yes/no) A ‘yes’ response to one or more items attracts the maximum scale score because undertaking one of the three actions is sufficient
9. Interacted with researchers	The extent to which individual policymakers contributed to academic research through collaboration, advisory roles or attending research fora; responses were in relation to the last 6 months	Items were based on Campbell et al.’s [11] seven item scale measuring involvement in research; items were collapsed (e.g. combining ‘collaboration on research write up’ with ‘authorship of a research publication’) with minor rewording; one item was adapted from Campbell et al.’s ‘links with researchers’ scale	6	Responses are on a 4-point adjectival scale ranging from ‘not at all’ (score = 1) to ‘more than twice’ (score = 4); items are summed to create a scale score (range 6 to 24)
*Research use – extent of use*				
10. Extent of research use	Use of research in each stage of the policy development process (agenda setting/scoping, development, implementation, evaluation) over the last 6 months	New items were written for this scale	4	Responses are on a 6-point adjectival scale ranging from ‘none’ (score = 1) to ‘extensive’ (score = 6); a ‘not applicable’ option is provided for stages not yet addressed (e.g. for a policy at the scoping stage, items about extent of use of research in policy evaluation are not applicable) The highest score across the four items is taken as the measure of the extent of research use (range 1 to 6)
*Research use – type of use*				
11. Conceptual research use	Use of research to understand an issue over the last 6 months	A new item was written for this measure	1	Binary response to individual items (yes/no)
12. Instrumental research use	Use of research to decide about content or direction of a policy or programme over the last 6 months	A new item was written for this measure	1	Binary response to individual items (yes/no)
13. Tactical research use	Use of research to persuade others to a point of view or course of action over the last 6 months	A new item was written for this measure	1	Binary response to individual items (yes/no)
14. Imposed research use	Use of research to meet organisational requirements over the last 6 months	A new item was written for this measure	1	Binary response to individual items (yes/no)

### Measurement properties of SEER

#### Sample and participant characteristics

**Fig. 3:**
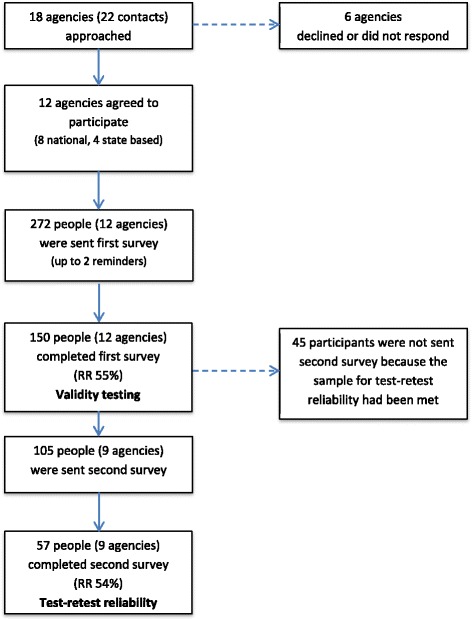
Recruitment of participants for SEER validation study

The flow of participants through the study, including response rate at each administration, is show in Fig. 3. From the first administration of SEER, 150/272 people (55%) from 12 agencies completed at least one SEER scale, contributing data to the validity analyses. Of the 150 respondents, 142 reported working on one or more policies or programmes in the prior 6 months (Table 2). The remaining eight respondents had not contributed to policy or programme development, so were administered the capacity scales only. SEER was administered a second time to 105 people from nine agencies (recruitment stopped when our target sample was reached), of whom 57 (54%) completed the survey, contributing data to the test-retest reliability analyses.

**Table 2:** Descriptive statistics for respondent characteristics

Characteristic (response options)	Freq. (%) or mean (SD)
Number of policies contributed to in the last 6 months^a^	
None^c^	8 (5%)
1 to 3	61 (41%)
More than 3	81 (54%)
Organisational tenure^a^	
0–1 years	28 (19%)
2–5 years	72 (48%)
6–10 years	33 (22%)
Over 10 years	11 (7%)
Did not respond	6 (4%)
Role tenure^a^	
0–1 years	45 (30%)
2–5 years	66 (44%)
6–10 years	24 (16%)
Over 10 years	9 (6%)
Did not respond	6 (4%)
Had received training in:^b^	
Evidence-based policy and programme development	68 (45%)
How to use research in policy and programme development	59 (39%)
Systematic reviews	57 (38%)
Percentage of time spent on:	
Policy development/design (mean (SD); n = 137)	11% (6) (IQR 7–16%)
Policy implementation (mean (SD); n = 126)	8% (5) (IQR 3–11%)
Policy evaluation (mean (SD); n = 127)	6% (6) (IQR 3–13%)

^a^Respondents were asked to select the option that best reflected their circumstance

^b^Respondents were asked to check all applicable boxes, therefore percentages do not sum to 100%. Denominator includes non-responders as well as those who had not received training

^c^This subset of respondents (n = 8) were administered the capacity scales, but not scales measuring research engagement or research use

Respondents most commonly reported working for between 2 and 5 years in their role (44%) and organisation (48%). About a third of respondents indicated that they had received training in the use of research in policy development (39%) or the use of systematic reviews (38%).

#### Interpretability of SEER: missing data and distribution of scores

The number of missing items from partially completed SEER scales was negligible; two respondents missed a single item each on different scales (Additional file 2). This provided indirect evidence that items and response options were interpretable. Of the 142 respondents administered scales from all three domains (capacity, research engagement, research use), 6 (4%) did not complete the four SEER scales measuring capacity. These scales were administered at the end of the survey, so non-completion may indicate respondent fatigue.

The distribution of scores (percentiles) for each item is presented in Additional file 2: Tables S2–S4 (see Additional file 4 for plots of distribution). The range of responses was restricted for two items in the scale measuring value individuals place on research (factor 1, 80% of respondents scored 4 or 5 on items 1.2 and 1.3; scale range 1–5), indicating a potential ceiling effect for these items, and one item in the ‘interacted with researchers’ scale (factor 9, 80% of respondents scored 1 or 2 on item 9.3; scale range 1–4), indicating a potential floor effect for this item.

#### Reliability of SEER: stability over time (test-retest analysis)

Estimates for test-retest reliability are presented in Table 3, with ICCs reported for continuous variables (capacity factors 1–4, research engagement factor 9, research use factor 10) and Cohen’s kappa coefficients reported for binary and ordinal variables (research engagement factors 5–8, research use factors 11–14).

**Table 3:** SEER test-retest reliability – Estimates of intraclass correlation coefficients (ICC) and Cohen’s kappa coefficient

Factor (items, scoring, possible range)	Test 1		Test 2		Test-retest^c^		Organisation^c^		Weighted^a^	
*Higher scores indicate greater capacity, more research engagement actions and use*	Mean (SD) [n] or response options	IQR or freq. (%)	Mean (SD) [n] or response options	IQR or freq. (%)	ICC	(95% CI)	ICC	(95% CI)	Kappa	(95% CI)
*Capacity – Predisposing factors*										
1. Value individual places on using research (7 items, summed, range 7–35)	29 (3.7) [143]	26–32	29 (3.8) [57]	27–31	0.59	(0.40–0.75)	0			
2. Confidence in using research (7 items, summed, range 7–35)	25 (6.0) [144]	22–28	24 (5.6) [57]	23–27	0.85	(0.76–0.91)	0.05	(0.00–0.47)		
3. Value organisation places on using research (5 items, summed, range 5–25)	19 (3.5) [144]	18–21	19 (3.7) [57]	16–21	0.76	(0.63–0.85)	0.13	(0.03–0.44)		
4. Tools and systems organisation has to support research use (7 items, summed,^d^ range 7–21)	14 (3.6) [144]	11–16	13 (3.6) [57]	10–16	0.70	(0.49–0.85)	0.48	(0.23–0.73)		
*Research engagement actions*										
5. Accessed synthesised research (two items, binary – yes/no^e^)	Yes No	112 (79) 30 (21)	Yes No	44 (79) 12 (21)					0.40	(0.10–0.69)
6. Accessed primary research (two binary items, summed, ordinal – 0, 1, 2)	0 1 2	20 (14) 27 (19) 95 (67)	0 1 2	6 (11) 10 (18) 40 (71)					0.49	(0.21–0.75)^b^
7. Appraised research (three binary items, summed, ordinal – 0, 1, 2, 3)	0 1 2 3	10 (8) 7 (6) 15 (12) 89 (74)	0 1 2 3	3 (6) 6 (12) 7 (14) 34 (68)					0.34	(0.04–0.69)^b^
8. Generated research (three binary items, coded yes if response to any item is yes, binary – yes, no)	Yes No	107 (76) 33 (24)	Yes No	39 (70) 17 (30)					0.39	(0.12–0.66)
9. Interacted with researchers (6 items, summed, range 6–24)	12 (4.7) [140]	8–15	11 (4.2) [55]	7–14	0.83	(0.66–0.92)	0			
*Research use*										
10. Extent of research use (4 items, choose item with highest score, range 1–6)^f^	5 (1.1) [140]	4–6	5 (1.2) [54]	4–6	0.65	(0.47–0.79)	0.14	(0.03–0.44)		
11. Conceptual research use (one item, binary – yes, no)	Yes No	125 (89) 15 (11)	Yes No	51 (93) 4 (7)					0.24	(−0.22 to 0.69)
12. Instrumental research use (one item, binary – yes, no)	Yes No	119 (85) 21 (15)	Yes No	50 (91) 5 (9)					0.49	(0.11–0.88)
13. Tactical research use (one item, binary – yes, no)	Yes No	117 (84) 23 (16)	Yes No	46 (84) 9 (16)					0.15	(−0.18 to 0.47)
14. Imposed research use (one item, binary – yes, no)	Yes No	66 (47) 74 (53)	Yes No	24 (44) 31 (56)					0.43	(0.18–0.67)

*ICC* intraclass correlation coefficient, *SD* standard deviation, *IQR* inter-quartile range, *n* sample size

^a^Weighted kappa using quadratic weights. Weights indicate the ‘degree’ of agreement. For example, with an ordinal variable with four values (0, 1, 2, 3), the weights are 0.8889, 0.5556, and 0 for a distance of one, two, and three apart, respectively. For example, if a participant’s factor score is 1 on the first application of the survey and 2 on the second application, they are 0.8889 in agreement. With no a prior rationale for a particular weighting scheme, this quadratic scheme is recommended [27]. In addition, a kappa statistic calculated using this weighting scheme will yield the same estimate as an ICC. ^b^Confidence interval for kappa calculated using bootstrapping. Bias corrected 95% confidence intervals were calculated from 1000 replicates. ^c^ICCs calculated from fitting a random effects model with two random effects (participant and organisation). This provides a measure of absolute agreement. ^d^Response options are: no (1), yes but limited (2), yes well developed (3), and I don’t know. For the purpose of psychometric testing, “I don’t know” was recoded as “no” rather than as a missing value. This assumes that “I don’t know” indicates that systems/tools are unlikely to function as a predisposing factor (i.e. motivating research engagement) if staff are unaware of their existence. ^e^Single score of ‘yes’ for factor if respondent answered ‘yes’ to either or both items. ^f^Score for this factor is the highest score from the four items (each reflecting a different stage of policy work: agenda setting/scoping, policy or programme development, policy or programme implementation, policy or programme evaluation). Respondents can respond ‘not applicable’ (0) for stages they have not covered, but at least one stage should be applicable so the minimum score for variable is 1

Test-retest reliability for the capacity scales was generally good, with ICCs above the conventional 0.7 threshold for three scales (factors 2–4, ICC range 0.70–0.85) and 0.59 for the scale measuring the ‘value individuals place on research’ (factor 1). The smaller ICC on the latter scale may partially be explained by the potential ceiling effect observed for two items on this scale because less variability between responses leads to smaller ICCs reflecting the potential for measurement error to mask differences in scores. Test-retest reliability was good for the research engagement scale measuring ‘interaction with researchers’ (factor 9, ICC 0.83) and acceptable for the ‘extent of research use’ scale (factor 10, ICC 0.65).

For each of the capacity scales, the correlation between responses within agencies was generally small (organisation ICCs ranging from 0 to 0.13) indicating that most of the variation in scores was explained by differences between individuals rather than differences between agencies. The exception was the ‘tools and systems’ scale (organisation ICC 0.48), for which a moderate correlation was observed between responses within the same agency. Consistent with our predictions, this indicates agreement between individuals within an agency about the extent to which their organisation has tools and systems to support research use.

Kappa coefficients were low for the research engagement scales (factors 5–8, weighted kappa ranged from 0.34 to 0.40) and for the binary measures of research use (factors 11–14, weighted kappa ranged from 0.15 to 0.49). The small observed kappas for binary measures was partially explained by the high prevalence of positive (yes) responses for most scales (prevalence indices (PI) ranging from 0.6 to 0.9; PI = 0 when ‘yes’ and ‘no’ are equally probable), except for ‘imposed research use’ where the relative probability of ‘yes’ and ‘no’ responses was similar (PI = 0.1) [44, 50].

#### Relations to other variables: convergence with similar measures and relation to outcomes

The pattern of correlations between SEER scale scores and TPB scale scores is shown in Table 4 (see Additional file 5 for confidence intervals and standard errors and Additional file 6 for plots of the association between SEER and TPB scores).

**Table 4:** Bivariate correlations between scores on SEER scales and theory of planned behaviour (TPB) scales

			TPB scales (number of items)		
	Behavioural intentions to use research (3)	Attitudes toward using research (4)	Subjective norms about using research (4)	Behavioural control – self efficacy^a^ (2)	Behavioural control – overall scale^b^ (4)
*Capacity – Predisposing factors*					
1. Value individual places on using research	0.311 (143)^d^	0.419 (138)^c^	0.373 (143)	0.149 (143)	0.084 (143)
2. Confidence in using research	0.292 (144)^d^	0.449 (138)^f^	0.211 (144)	0.671 (144)^c^	0.457 (144)^c^
3. Value organisation places on using research	0.128 (144)^d^	0.185 (138)	0.541 (144)^c^	0.137 (144)	0.062 (144)
4. Tools and systems organisation has to support research use	0.174 (144)^d^	0.223 (138)	0.480 (144)^f^	0.326 (144)	0.167 (144)
*Research engagement actions*					
5. Accessed synthesised research	0.201 (136)^e^	0.150 (131)^d^	0.400 (136)^d^	0.230 (136)^d^	0.184 (136)
6. Accessed primary research	0.189 (136)^e^	0.148 (131)^d^	0.221 (136)^d^	0.137 (136)^d^	0.176 (136)
7. Appraised research	0.310 (119)^e^	0.231 (116)^d^	0.289 (119)^d^	0.299 (119)^d^	0.107 (119)
8. Generated research	0.173 (136)	0.175 (131)	0.150 (136)	0.154 (136)	0.179 (136)
9. Interacted with researchers	0.169 (136)	0.145 (131)	0.300 (136)	0.232 (136)	0.351 (136)
*Research use*					
10. Extent of research use	0.302 (136)^e^	0.313 (131)^d^	0.355 (136)^d^	0.278 (136)^d^	0.231 (136)
11. Conceptual research use	0.104 (136)^e^	0.216 (131)^d^	0.204 (136)	0.057 (136)^d^	0.059 (136)
12. Instrumental research use	0.178 (136)^e^	0.141 (131)^d^	0.239 (136)^d^	0.162 (136)^d^	0.153 (136)
13. Tactical research use	0.204 (136)^e^	0.083 (131)	0.088 (136)	0.143 (136)^d^	0.052 (136)
14. Imposed research use	0.184 (136)	0.182 (131)	0.373 (136)^d^	0.160 (136)	0.089 (136)

^a^Items 9 and 10 of TPB measure

^b^Items 9, 10, 11 and 12 of TPB measure. Item 11 was recoded so that higher scores consistently reflect greater control over research use

^c^Indicates the construct measured by SEER and the corresponding TPB scale is similar (i.e. convergence of scores is expected)

^d^Indicates where one scale measures a predictor of research use (i.e. SEER capacity scales; TPB ‘attitudes’, TPB ‘social norms’, TPB ‘behavioural control’ scales) and the other measures an outcome, being either intention to use research (TPB ‘behavioural intention’ scale) or self-reported behaviour

^e^Indicates where we predicted positive, small to moderate correlations between TPB-’behavioural intention’ and SEER scales measuring self-reported behaviour

^f^Indicates where no predictions were made, but where positive, moderate correlations (> 0.4) were observed between scores on SEER and TPB scales

##### Convergence with similar measures

Cells marked with the subscript ‘c’ are those where the construct measured by SEER and the corresponding TPB scale is similar (i.e. convergence of scores is expected). We predicted a positive, moderate to large correlation (0.4–0.8) [27] between scores on these scales, meaning that we expected that people with higher scores on the SEER scale would have higher scores on the corresponding TPB scale. A smaller correlation was predicted with the overall TPB ‘behavioural control’ scale score, because the scale includes items about whether the individual feels the decision to use research is within their control (a construct not measured by SEER) in addition to items measuring self-efficacy (similar to the SEER ‘confidence’ scale). Our predictions were supported for all three SEER capacity scales, providing evidence of convergence between SEER scores and scores on scales measuring similar concepts.

##### Relation between research-use predictors and outcomes

Cells marked with the subscript ‘d’ are those where one scale measures a predictor of research use (i.e. SEER capacity scales; TPB ‘attitudes’, TPB ‘social norms’, TPB ‘behavioural control’ scales) and the other measures an outcome, being either intention to use research (TPB ‘behavioural intention’ scale) or self-reported behaviour (SEER research engagement and research use scales). We hypothesised that scores on the SEER ‘value of research’ scale would predict TPB ‘behavioural intention’ (i.e. people that valued research more highly were expected to have a stronger intention to use research), resulting in a positive, and moderate to large correlation. Positive, small to moderate correlations were predicted between scores on all other scales marked by the superscript 'd'. The observed correlations between SEER capacity scale scores and TPB ‘behavioural intention’ were smaller than expected, which might partly be explained by a ceiling effect observed for the TPB ‘behavioural intention’ scale (80% of respondents scored 6–7; scale range 1–7; Additional file 4: Scatter plots). This restricted range of responses is likely to lead to underestimation of the correlation between TPB ‘behavioural intention’ and all SEER scale scores [51]. Small correlations were observed between scores on TPB scales measuring predictors of research use and the SEER ‘extent of research use’ scale. These were consistent with our predictions, but smaller in magnitude. Correlations between scores on TPB scales measuring predictors of research use and the SEER research engagement and SEER type of research use scales were small in magnitude, with no clear pattern between observed correlations and our predictions.

Cells marked with the subscript ‘e’ are where we predicted positive, small to moderate correlations between TPB ‘behavioural intention’ and SEER scales measuring self-reported behaviour. No clear pattern emerged between observed correlations and our predictions.

Cells marked with the subscript ‘f’ are those for which no predictions were made, but where positive, moderate correlations (>0.4) were observed between scores on SEER and TPB scales. Higher scores on the SEER ‘confidence’ scale were associated with higher scores on the TPB scale measuring ‘attitudes’, and a correlation of similar magnitude was seen between the SEER scale measuring ‘tools and systems’ and the TPB scale measuring ‘subjective norms’ (how much a person feels social pressure to use research [52]).

#### Internal structure of SEER capacity scales

Table 5 presents the CFA conducted to assess the proposed measurement model for the SEER capacity scales. The second panel shows results from CFA models in which each latent construct (factor) was fitted separately, while the third panel shows how the coefficients changed when we fitted the full model, allowing for correlation among the latent constructs. The standardised loadings provide an estimate of the shift in the item score, in terms of standard deviation units, for a one standard deviation shift on the latent factor. The standardised loadings ranged from 0.43 to 0.72 for ‘value individual places on using research’; 0.69 to 0.87 for ‘confidence’; 0.56 to 0.88 for ‘value organisation places on using research’; and 0.54 to 0.67 for ‘tools and systems’. All factor loadings were highly statistically significant (all *P* < 0.001). The loadings were generally smaller for ‘tools and systems’, which was likely due to downward bias arising from the use of ordinal items with only three values (response options).

**Table 5:** Estimates from confirmatory factor analysis models

	Separate models^a^			Full model^a^			Modified model (removal of item 2.3)^a^		
Factor/items	b	(95% CI)	*P* value	b	(95% CI)	*P* value	b	(95% CI)	*P* value
1. *Value individual places on using research*									
1.1. Identify issues that require a policy or programme response	0.59	(0.50–0.68)	< 0.001	0.60	(0.52–0.69)	< 0.001	0.60	(0.52–0.69)	< 0.001
1.2. Understand how to think about issues	0.60	(0.47–0.72)	< 0.001	0.59	(0.48–0.70)	< 0.001	0.59	(0.48–0.70)	< 0.001
1.3. Decide about content or direction of a policy or programme	0.69	(0.56–0.82)	< 0.001	0.71	(0.59–0.82)	< 0.001	0.70	(0.59–0.82)	< 0.001
1.4. Persuade others to a point of view or course of action	0.52	(0.40–0.65)	< 0.001	0.55	(0.42–0.67)	< 0.001	0.55	(0.43–0.68)	< 0.001
1.5. Design the implementation or evaluation strategy for a policy or program	0.75	(0.59–0.90)	< 0.001	0.72	(0.55–0.90)	< 0.001	0.72	(0.55–0.90)	< 0.001
1.6. Monitor implementation or evaluate the impact of a policy or program	0.66	(0.50–0.82)	< 0.001	0.63	(0.47–0.80)	< 0.001	0.64	(0.47–0.80)	< 0.001
1.7. Meet organisational requirements to use research	0.41	(0.17–0.66)	0.001	0.43	(0.18–0.67)	0.001	0.43	(0.18–0.67)	0.001
2. *Confidence in using research*									
2.1. Find research to inform policy or programme development	0.83	(0.75–0.91)	< 0.001	0.82	(0.74–0.91)	< 0.001	0.76	(0.65–0.88)	< 0.001
2.2. Evaluate the quality of research	0.88	(0.84–0.93)	< 0.001	0.87	(0.81–0.92)	< 0.001	0.79	(0.69–0.89)	< 0.001
2.3. Interpret the results of research	0.83	(0.73–0.92)	< 0.001	0.81	(0.70–0.92)	< 0.001			
2.4. Apply research to policy or programme development	0.81	(0.67–0.94)	< 0.001	0.81	(0.68–0.95)	< 0.001	0.82	(0.70–0.94)	< 0.001
2.5. Design evaluations of policies or programmes	0.81	(0.70–0.91)	< 0.001	0.81	(0.71–0.92)	< 0.001	0.85	(0.79–0.90)	< 0.001
2.6. Commission research to support policy or programme development	0.67	(0.51–0.84)	< 0.001	0.69	(0.52–0.86)	< 0.001	0.76	(0.64–0.88)	< 0.001
2.7. Partner with researchers to generate research	0.71	(0.55–0.86)	< 0.001	0.72	(0.56–0.88)	< 0.001	0.79	(0.69–0.89)	< 0.001
3. *Value organisation places on using research*									
3.1. Leaders believe it is important to use research in policy or programme development	0.87	(0.81–0.94)	< 0.001	0.85	(0.75–0.95)	< 0.001	0.85	(0.76–0.95)	< 0.001
3.2. It is expected that research will be used in policy or programme development	0.90	(0.84–0.96)	< 0.001	0.88	(0.77–0.99)	< 0.001	0.88	(0.77–0.99)	< 0.001
3.3. Generation of new research to inform policy or programme development is encouraged	0.62	(0.42–0.82)	< 0.001	0.65	(0.46–0.83)	< 0.001	0.65	(0.46–0.83)	< 0.001
3.4. It is expected that policies/programmes will be evaluated	0.54	(0.28–0.80)	< 0.001	0.56	(0.33–0.78)	< 0.001	0.56	(0.33–0.78)	< 0.001
3.5. Interaction or collaboration with researchers or research organisations is encouraged	0.67	(0.53–0.80)	< 0.001	0.71	(0.56–0.86)	< 0.001	0.71	(0.56–0.85)	< 0.001
4. *Tools and systems*									
4.1. Has processes for policy or programme development that provide guidance on how research should be used	0.61	(0.50–0.71)	< 0.001	0.59	(0.48–0.70)	< 0.001	0.59	(0.48–0.70)	< 0.001
4.2. Has systems that encourage leaders to support use of research	0.67	(0.57–0.78)	< 0.001	0.67	(0.57–0.76)	< 0.001	0.67	(0.58–0.76)	< 0.001
4.3. Provides access to training in using research in policy or programme development	0.52	(0.29–0.75)	< 0.001	0.54	(0.33–0.75)	< 0.001	0.54	(0.33–0.74)	< 0.001
4.4. Has the resources needed to access research	0.53	(0.31–0.76)	< 0.001	0.55	(0.34–0.76)	< 0.001	0.54	(0.33–0.75)	< 0.001
4.5. Has established methods for commissioning reviews of research	0.65	(0.47–0.84)	< 0.001	0.64	(0.46–0.82)	< 0.001	0.64	(0.46–0.82)	< 0.001
4.6. Has documented processes for how policies or programmes should be evaluated	0.60	(0.42–0.77)	< 0.001	0.60	(0.43–0.76)	< 0.001	0.60	(0.45–0.76)	< 0.001
4.7. Has existing relationships, or established methods for engaging, with research organisations	0.65	(0.50–0.80)	< 0.001	0.64	(0.47–0.81)	< 0.001	0.64	(0.48–0.80)	< 0.001

^a^b standardised loading

The model fit was assessed by the SRMSR index, which yielded a value of 0.082. This value was above the suggested threshold of 0.08, indicating that the model may not be fitting well (but below 0.1 which is the threshold for a poor fit) [53]. Examination of the modification indices suggested that the model would be improved by adding paths between item 2.3 ‘Interpret the results of research’ and the factors ‘value individual places on using research’, ‘value organisation places on using research’, and ‘tools and systems’. This suggested that item 2.3 may not be particularly discriminating, despite the item having face validity as an indicator of individual confidence rather than as an organisational attribute. We therefore dropped item 2.3 from the model (Table 5). The standardised loadings estimated from the modified model were similar to the full model. There was a slight improvement in model fit, as measured by the SRMSR, which reduced to 0.076 (i.e. below the 0.08 threshold). The coefficient of determination was 0.999 for both models.

Table 6 shows the correlation among scales (latent factors) for the full and modified models, together with the means, standard deviations, and alpha coefficients (estimating scale internal consistency) for scales in the full model. All four scales demonstrated adequate internal consistency reliability (alpha from 0.80 to 0.92). Small to moderate correlations were observed between most factors in both the full and modified models (0.20 to 0.68), suggesting that the scales measured related but distinct concepts (a further test of validity). The largest correlation observed was between the ‘value organisation places on using research’ and organisational ‘tools and systems’ factors (0.68), supporting the hypothesis that organisations that value research more highly are more likely to invest in tools and systems to use research. The smallest correlation was between ‘confidence’ and ‘value organisation places on using research’.

**Table 6:** Descriptive statistics, Cronbach’s alpha coefficients, and correlations between factors in full SEER measurement model and modified model^a^

	Full model				Factor correlations (95% CI) *P* value		
Factor (score range)	Mean	SD	α	1	2	3	4
1. Value individual places on using research (7–35)	29	3.7	0.80		0.26 (0.18–0.35) < 0.001^b^	0.38 (0.17–0.59) < 0.001^b^	0.25 (0.06–0.44) 0.010^b^
2. Confidence in using research (7–35)	25	6.0	0.92	0.20 (0.10–0.31) <0.001		0.15 (0.00–0.29) 0.047^b^	0.43 (0.28–0.59) < 0.001^b^
3. Value organisation places on using research (5–25)	16	3.5	0.85	0.38 (0.17–0.60) 0.001	0.12 (−0.03 to 0.26) 0.121		0.68 (0.55–0.80) < 0.001^b^
4. Tools and systems organisation has to support research use (7–21)	14	3.6	0.80	0.25 (0.06–0.44) 0.010	0.38 (0.19– 0.56) < 0.001	0.68 (0.55–0.81) < 0.001	

^a^Item 2.3 was dropped from the modified model

^b^Modified model

## Discussion

Capacity building strategies have been widely used to increase the use of research in health policy. However, an absence of well-validated measures of individual capacity has hampered efforts to identify priorities for capacity building and evaluate the impact of different strategies. We aimed to address this gap by developing SEER, a tool designed as a self-report measure of individual policymakers’ capacity to engage with and use research. SEER complements previously reported measures of organisational capacity to use research [2, 24, 25], with the aim of enabling more comprehensive assessment of capacity to engage with and use research at different organisational levels. Here, we discuss our main findings about the measurement properties of SEER, consider priorities for further testing and refinement of the scales, and conclude by outlining the ways in which SEER could be used by researchers and policymakers.

### What we know about the measurement properties of SEER

#### Capacity scales

Our findings provide initial evidence supporting the use of SEER for assessing individual capacity in policy agencies. We found evidence that scores on SEER capacity scales converge with scores on scales measuring similar concepts (based on bivariate correlations with TPB scales), and that the four capacity scales measure related but distinct concepts (based on correlation patterns from the factor analysis). There is empirical evidence from our factor analysis that the items in each of the four scales relate as predicted to concepts in the measurement model derived from the SPIRIT Action Framework. The scales demonstrate adequate reliability for instruments used in research based on conventional thresholds for test-retest and internal consistency reliability (ICC and alpha coefficients > 0.7 for most scales) [27]. Assessment of the distribution of scale scores (notably, possible ceiling effects for two ‘value an individual places on using research’ items) and the modified measurement model (demonstrating improved fit following removal of one ‘confidence’ item) suggests refinements that may enhance the measurement properties of SEER. However, the items in question have face validity without obvious redundancy. Since the version of SEER we tested appears to have acceptable properties overall, we do not recommend changes without further testing.

#### Research engagement and research use scales

The continuous scale measuring ‘extent of research use’ shows promise as a global indicator of research use. Small associations were observed with three TPB scales measuring predictors of research use, and the scale had reasonable test-retest reliability. A valid single item, self-report measure of research use would be a useful inclusion in a battery of items used for routine monitoring of the impact of capacity building strategies, so this measure warrants further evaluation. The ‘interacting with researchers’ scale demonstrated acceptable test-retest reliability, but our predictions for how scores on this scale would relate to those on TPB scales were not upheld. No clear pattern emerged from our tests of the relation between other SEER measures of research engagement and use, and the TPB scales measuring predictors of research. Several factors may explain our findings. First, these were binary or ordinal measures where the prevalence of ‘yes’ responses was typically high. As such, the measures may not discriminate well between individuals, resulting in poorer reliability (as observed in low kappas) and smaller correlations with related variables. Second, these are proxy or indirect measures of research use behaviours. Evaluation of self-report measures of behaviour in other contexts has shown that bias (systematic error) associated with such measures weakens observed associations with related measures [39]. Finally, the different types of research use (e.g. conceptual, tactical) reflect abstract behaviours that are difficult to describe, further complicating their measurement.

### Priorities and opportunities for further testing and refinement of SEER

#### Capacity scales

Two principal lines of testing are needed to confirm and refine the properties the SEER capacity scales. First, establishing that the capacity scales predict research-use outcomes and discriminate between different levels of capacity is key to confirming the suitability of SEER for evaluation. Such tests are challenging because of the paucity of objective measures of research use for testing predictive validity, and of gold-standard measures of capacity required to discriminate between groups. Second, replication of the factor analysis in a larger sample is needed to provide additional support for the proposed measurement model, and identify refinements that enhance the reliability or validity of SEER. Both lines of testing could be undertaken using data from the SPIRIT study. SPIRIT includes an objective measure of research engagement and use (SAGE) [38] that would enable testing of the predictive validity of SEER, and a measure of organisational tools and systems (ORACLe) [25] that could be used to differentiate between agencies exhibiting different levels of support.

#### Research engagement and use

Some additional testing is required for the ‘extent of research use’ and ‘interaction with researchers’ scales, while the binary and ordinal measures require more substantive refinement and evaluation. As with the SEER capacity scales, the ‘extent of research use’ and the ‘interaction’ scales could be validated using SPIRIT data from SAGE, the objective measure of research engagement and use. Extending our factor analytic model to include these latent factors and indicators would provide a further test of validity. One option for measuring research engagement using the current items might be to combine scores for the five research engagement actions to calculate a score for the higher order construct ‘research engagement’. If the goal of measurement is to assess overall research engagement (as opposed to specific actions), then this approach would have face validity. Extending the factor analytic model to include this variable would provide a test of whether the items measure a higher order construct. In contrast, if the goal is to measure specific engagement actions, then rescaling these measures to generate continuous variables is likely to be a better approach. The latter would require re-wording and re-testing.

### Ways in which SEER could be used for research and practice in policy agencies

SEER provides a practical tool that we envisage will have three main applications: in policy agencies seeking to assess or develop their capacity for using research; in research investigating how capacity and capacity building strategies influence research use; and at the nexus where policymakers and researchers partner.

#### Application of SEER in policy agencies

SEER was conceived as a tool for monitoring and providing feedback about capacity in policy agencies. It was designed for use in ongoing efforts to strengthen research use (continuous improvement) and during more transformative change. In the SPIRIT trial, feedback from SEER was used to stimulate discussion in policy agencies about their priorities for capacity building, thus guiding intervention selection. During the testing of SEER, one agency discussed using their SEER data in a baseline assessment prior to introducing structures to support research use, while another saw potential to benchmark the maturity of their capacity against other agencies. SEER may have utility for all these purposes and, with further validation, may be useful for assessing capacity at individual, team, and unit level or in relation to development of specific policy.

#### Application of SEER in research

SEER is one of three instruments designed to measure concepts in the SPIRIT Action Framework [36]. As such, it underpins the framework’s structured approach to designing and testing strategies intended to increase the use of research in policy. The SPIRIT trial illustrates this approach wherein SEER is used to examine the extent to which individual capacity mediates the effects of a capacity building strategy on research engagement and research use outcomes (measured by SAGE) [37]. SEER could also be used to investigate which aspects of capacity most influence the use of research and those most amendable to change. Such research is required to identify areas where investment in capacity building is most likely to have an impact. It is also needed to confirm, refine or refute the content of the SPIRIT Framework, which may ultimately lead to changes in the content of SEER.

#### Application of SEER at the nexus between policy and research

Perhaps the greatest value of SEER lies in its potential to help facilitate and maximise the value of partnerships between policymakers and researchers. SEER data could inform discussions about partnering to build capacity and where best to invest resources. In turn, SEER could be used to monitor the impact of the partnership, providing formative data from which to optimise intervention outcomes. In this context, capacity can be more feasible to measure than distal outcomes like inclusion of research in policy. Change can be detected in shorter time frames and may be more directly attributable to the partnership.

### Strengths and limitations

To our knowledge, this study reports the most comprehensive assessment to date of a measure of individual capacity to engage with and use research. SEER is one of the first measures to assess the broad range of factors reported to influence an individual policymakers’ use of research. Development of SEER and the SPIRIT Action Framework, on which SEER was based, occurred in parallel, so those evaluating the content of SEER had working knowledge of the domains and concepts to be measured. Together with pilot testing, this approach helped maximise the validity of the content of SEER. Our testing was conducted in multiple agencies involved in a wide spectrum of policy and programme work, increasing the generalisability of our findings.

The study has limitations. This first test of SEER provides initial evidence about its reliability and validity, but further testing is needed to support the use of SEER for all purposes for which it was developed. As with all measures, replication of testing across settings and policy contexts is required to strengthen the evidence supporting SEER. It was not feasible to recruit a random sample of policy agencies or policymakers within agencies. Although most statistical tests assume a random sample, the impact of our sampling approach is uncertain. The response rate for the first and second administrations of SEER was 55% and 54%, respectively. This is typical of surveys of this type among professional groups [54], but may introduce selection bias if those who respond differ systematically from non-responders. We did not have data about non-responders to determine if there were any characteristics for which there were systematic differences.

### Future research

The preceding discussion covers two interdependent areas of future research; the first aimed at ensuring SEER is a valid measure, and the second aimed at understanding and developing capacity for using research in policy work. Application of SEER in research has potential to address questions relating to both aims. Our immediate priorities are to use data from the SPIRIT trial to examine whether SEER predicts objectively measured research-use outcomes, and to replicate and extend the factor analysis. These tests will provide further evidence about the validity of the SEER individual capacity scales, confirming or refuting the need for refinement. Administering SEER with a measure that enables differentiation of organisations with different capacity for using research (i.e. tools and systems, value placed on research) would provide data to test whether SEER scales measuring individual perceptions of an organisation can discriminate between groups. Combining data from participant organisations in SPIRIT and our measures testing may enable such a test. Work is also required to refine and test the measures of research engagement and use, with one option being to re-word and re-scale items so that all concepts are measured as continuous variables. Finally, testing and refinement of SEER will need to be responsive to accumulating evidence that confirms, refutes or refines the theories on which it is based.

## Conclusion

To our knowledge, this paper reports the most comprehensive attempt to date to develop and test a measure of individual capacity to engage with and use research in policy development. Based on the SPIRIT Action Framework, SEER reflects contemporary understanding of individual-level factors that may influence the use of research in policy. Our initial tests of SEER provide evidence that the four individual capacity scales may be used in policy settings to examine current capacity and identify areas for capacity building. These scales complement previously reported measures of organisational capacity, enabling researchers and policy agencies to assess factors influencing research use at different organisational levels. Further research is required to confirm the suitability of these scales for other applications, and to improve the pragmatic self-report measures of research engagement and use included in SEER. Using SEER in intervention and observational studies examining the relation between capacity, research engagement actions, and research use will help provide this evidence.

## Additional files

Additional file 1: Analysis of existing self-report measures published prior to development of SEER. (DOCX 49 kb)
Additional file 2: Tables S2–S3. SEER items, scoring and descriptive statistics. (DOCX 45 kb)
Additional file 3: Theory of planned behaviour model and instrument. (DOCX 36 kb)
Additional file 4: Plots showing distribution of SEER scale scores. (PDF 204 kb)
Additional file 5: Excel file reporting correlation between SEER and theory of planned behaviour scale scores with confidence intervals and standard errors. (XLSX 10 kb)
Additional file 6: Plots showing association between SEER and theory of planned behaviour scale scores. (PDF 189 kb)
