# Supplementary material for: Development and validation of SEER (Seeking, Engaging with and Evaluating Research): a measure of policymakers’ capacity to engage with and use research
Source: Health Res Policy Syst. 2017 Jan 17;15:1. doi: 10.1186/s12961-016-0162-8 (PMC5240393; doi:10.1186/s12961-016-0162-8)
Supplement: Additional file 1: — Analysis of existing self-report measures published prior to development of SEER. (DOCX 49 kb) [file 12961_2016_162_MOESM1_ESM.docx]

## Additional file 1. Existing measures

## Analysis of existing self-report measures published prior to development of SEER

Prior to developing SEER, we searched for quantitative measures of concepts in the SPIRIT Action Framework by reviewing literature and consulting with researchers internationally recognised for their expertise in evidence informed-policy. The measures identified fell into three categories: (i) comprehensive measures designed to measure multiple aspects of capacity in policy contexts; (ii) comprehensive measures designed for other contexts (e.g. for healthcare providers as reviewed by Squires and colleagues [[1](#_ENREF_1)]), and (iii) measures of single concepts (factors) in the SPIRIT Action Framework (e.g. measures of interaction between policy makers and researchers).

To confirm the need for SEER, self-report instruments in the first category were assessed against the following criteria (i) coverage of SPIRIT domains, (ii) suitability of item wording and context for measuring individual policy makers’ perceptions and actions (level of measurement), (iii) evidence that the instrument supports valid and reliable measurement for its stated purpose, and (iv) feasibility and acceptability of repeated administration (as may be required in a randomised trial or when the measure is applied in practice). Instruments in the second two categories were examined for their potential to be adapted or as a source of items for SEER (see ‘METHODS’).

## Results of assessment of comprehensive measures of capacity for research use in policy contexts

Table S1 summarises the characteristics of measures similar to SEER and their attributes in relation to our four criteria. Like SEER, the CIHR measure [[2](#_ENREF_2)] and the instrument adapted from it by Oxman et al [[3](#_ENREF_3)] were designed to help agencies assess and develop their capacity to use research. Both instruments are quantitative, but were designed to obtain a consensus measure of organisational-capacity rather than individual-capacity. These instruments ask about organisational capacity for undertaking the research engagement actions identified in SPIRIT (e.g. accessing and appraising research), but not individual confidence or values in relation to these actions.

We identified one potentially relevant measure of individual capacity to use research; an instrument based on the theory of planned behaviour (TPB measure) [[4](#_ENREF_4)] [Table S1]. This instrument measures intention to use research and factors that predict intention, namely: attitudes (whether a person is in favour of using research), subjective norms (the social pressure a person feels to use research), and behavioural control (whether a person feels control over the decision to use research and efficacy for using research). The measure is not suitable for operationalising SPIRIT because the predictors of intention are not examined in relation to specific research engagement actions. Nor are policy makers asked about actions taken to engage with research. However, the measure has evidence supporting validity of its content and reliability (Table S1) [[4](#_ENREF_4)], and scale scores are expected to relate to SEER scores, making the measure suitable for examining the validity of SEER.

Table S1. Description and assessment of instruments covering domains measured by SEER

|  | CIHR – Is research working for you? (Kothari 2009) | SUPPORT tool  (Oxman 2011) | TPB measure  (Boyko 2011) |
| --- | --- | --- | --- |
| **Description** |  |  |  |
| Purpose | Diagnostic: self-assessment of capacity for use of research in health services management and policy agencies | Diagnostic: self-assessment of capacity to use research evidence in health policy | Evaluative: measure of intention to use research for assessing effectiveness of knowledge translation and exchange strategies |
| Stated domains (number of items) | Capacity (skills, processes, structures) to acquire (7), assess (4), and adapt (4) research; capacity and culture to apply research (12). | Culture and values (6); professional development (6); capacity to prioritise (5), acquire (5) and appraise research (5); capacity to inform decisions with research (5); capacity to monitor/evaluate policy (5). | Behavioural intention to use research (3); predictors of behavioural intention - attitudes toward using research (4), social norms (4), behavioural control (4) |
| **Coverage of domains in the SPIRIT Action Framework** | | | |
| Capacity |  |  |  |
| Value individual places on using research | Not covered | Not covered | Partial: attitudes (similar to individual values) measured in relation to use of research in general, not specific engagement actions |
| Confidence in knowledge and skills | Not covered | Not covered | Partial: self-efficacy (similar to confidence) measured in relation to use of research in general, not specific engagement actions |
| Value organisation places on using research | Covered | Covered | Partial: social norms (similar to organisational value) measured in relation to use of research in general, not specific engagement actions |
| Tools and systems | Covered | Covered | Not covered |
| Research engagement actions | Not covered | Not covered | Not covered |
| Research use | Not covered | Not covered | Partial: measures intention to use research (behavioural intention), not a retrospective measure of actual behaviour (extent or type of research use). |
| **Level of measurement** |  |  |  |
|  | Organisation – consensus across individual responses. Items framed in relation to the organisation’s capacity, culture, and practices. | Organisation – consensus across individual responses. Items framed in relation to the organisation’s capacity, culture, and practices. | Individual policy makers. Items framed in relation to the individual’s attitudes, beliefs and intentions. |
| **Evidence of validity and reliability*** | | | |
| Content | Yes: Based on literature review, content developed with input from potential respondents in focus groups [[2](#_ENREF_2), [5](#_ENREF_5)] | Yes: Based on CIHR and Lavis 2006 frameworks; adapted from CIHR tool with iterative revision based on workshops with ‘various groups’ [[3](#_ENREF_3)] | Yes: Based on theory of planned behaviour (TPB); content developed according to guidance for TPB measures with expert input, pilot testing [[4](#_ENREF_4)] |
| Construct (relations to other variables, structure) | Limited testing: Discrimination between groups expected to differ (single study, 30 groups) [[5](#_ENREF_5)] | Limited testing: Discrimination between groups expected to differ (single study, N=57 but in healthcare setting) [[6](#_ENREF_6)] | Not tested: Ongoing studies may generate evidence of whether measure predicts outcomes, discriminates between groups. |
| Reliability | Not tested | Limited testing: Internal consistency (single study, N=57) [[6](#_ENREF_6)] | Yes: Internal consistency (but not factor analysis); test re-test (Pearson correlation coefficients [r]; G coefficients) (single study, N=62): behavioural intention (r=0.29), predictors of intention (r 0.67-0.77) [[4](#_ENREF_4)]. Authors recommend administering the instrument twice to discriminate between respondents. |
| **Administration and interpretation** | | | |
|  | Survey administered to representatives across levels of the organisation, then group discussion to obtain a consensus score. | Implied, but not stated, that survey administered to (multiple) individuals. Responses used as a ‘scorecard’ to stimulate discussion and obtain a consensus score. | Self-report, administered to individuals. |
| **Variants and instances of use*** | | | |
|  | SURE/SUPPORT papers [[3](#_ENREF_3)]; Outcome measure for evaluation of an evidence briefing service (protocol for before-after study) [[7](#_ENREF_7)]. Both papers report modifications of the instrument that are likely to impact on measurement properties. | Also available as SURE guides: <http://global.evipnet.org/SURE-Guides/source/about.html>  Limited test of validity and reliability (as above) [[6](#_ENREF_6)]. Protocol for evaluation of an evidence briefing service [[7](#_ENREF_7)] cites use of a modified version of the SURE tool, but items appear more similar to the CIHR tool. | Outcome measure for evaluation of an evidence service (protocol for randomised trial; trial stopped due to failure to recruit) [[8](#_ENREF_8), [9](#_ENREF_9)]. Evaluation of deliberative dialogues/ evidence briefs (descriptive statistics reported) [[10](#_ENREF_10), [11](#_ENREF_11)]. Outcome measure for evaluation of an evidence briefing service (protocol for before-after study; modification of measure) [[7](#_ENREF_7)]. |

*Includes information identified following citation searches for published papers reporting use or testing of the instrument.

## References

1. Squires JE, Estabrooks CA, O'Rourke HM, Gustavsson P, Newburn-Cook CV, Wallin L: **A systematic review of the psychometric properties of self-report research utilization measures used in healthcare**. *Implement Sci* 2011, **6**(1):83.

2. Kothari A, Edwards N, Hamel N, Judd M: **Is research working for you? validating a tool to examine the capacity of health organizations to use research**. *Implement Sci* 2009, **4**:46.

3. Oxman AD, Vandvik PO, Lavis JN, Fretheim A, Lewin S: **SUPPORT Tools for evidence-informed health Policymaking (STP) 2: Improving how your organisation supports the use of research evidence to inform policymaking**. *Health Res Policy Syst* 2009, **7 Suppl 1**:S2.

4. Boyko J, Lavis J, Dobbins M, Souza N: **Reliability of a Tool for Measuring Theory of Planned Behaviour Constructs for use in Evaluating Research Use in Policymaking**. *Health Research Policy and Systems* 2011, **9**(1):29.

5. Thornhill J, Judd M, Clements D: **CHSRF Knowledge Transfer: (Re)Introducing the Self-Assessment Tool That Is Helping Decision-Makers Assess Their Organization's Capacity to Use Research**. *Healthcare Quarterly* 2009, **12**(1):22-24.

6. Catallo C, Sidani S: **The self-assessment for organizational capacity instrument for evidence-informed health policy: Preliminary reliability and validity of an instrument**. *Worldviews on Evidence-Based Nursing* 2014, **11**(1):35-45.

7. Wilson PM, Farley K, Thompson C, Chambers D, Bickerdike L, Watt IS, Lambert M, Turner R: **Effects of a demand-led evidence briefing service on the uptake and use of research evidence by commissioners of health services: protocol for a controlled before and after study**. *Implement Sci* 2015, **10**(1):7.

8. Lavis J, Wilson M, Grimshaw J, Haynes R, Hanna S, Raina P, Gruen R, Ouimet M: **Effects of an evidence service on health system policymakers' use of research evidence: A protocol for a randomized controlled trial**. *Implementation Science* 2011, **6**(1):51.

9. Wilson M, Grimshaw J, Haynes R, Hanna S, Raina P, Gruen R, Ouimet M, Lavis J: **A process evaluation accompanying an attempted randomized controlled trial of an evidence service for health system policymakers**. *Health Research Policy and Systems* 2015, **13**(1):78.

10. Lavis JN, Boyko JA, Gauvin FP: **Evaluating deliberative dialogues focussed on healthy public policy**. *BMC Public Health* 2014, **14**:1287.

11. Moat KA, Lavis JN, Clancy SJ, El-Jardali F, Pantoja T, Knowledge Translation Platform Evaluation study team: **Evidence briefs and deliberative dialogues: perceptions and intentions to act on what was learnt**. *Bull World Health Organ* 2014, **92**(1):20-28.
