# Supplementary material for: Development and validation of SEER (Seeking, Engaging with and Evaluating Research): a measure of policymakers’ capacity to engage with and use research
Source: Health Res Policy Syst. 2017 Jan 17;15:1. doi: 10.1186/s12961-016-0162-8 (PMC5240393; doi:10.1186/s12961-016-0162-8)
Supplement: Additional file 2: Tables S2–S3. — SEER items, scoring and descriptive statistics. (DOCX 45 kb) [file 12961_2016_162_MOESM2_ESM.docx]

Table S2. Item descriptive statistics for SEER capacity scales (predisposing factors)

| Factors | Items | n | mean | SD | Distribution (percentile) | | | | | Response options (score)* |
| --- | --- | --- | --- | --- | --- | --- | --- | --- | --- | --- |
|  |  |  |  |  | p10 | p25 | p50 | p75 | p90 |  |
| 1. **Value individual** places on using research | It is valuable to use research in policy or program work to: |  |  |  |  |  |  |  |  |  |
|  | - 1. Identify issues that require a policy or program response | 144 | 4 | 0.8 | 3 | 4 | 4 | 5 | 5 | 5 point adjectival scale; not at all valuable (1) to very valuable (5) |
|  | - 1. Understand how to think about issues | 144 | 4 | 0.7 | 4 | 4 | 4 | 5 | 5 |  |
|  | - 1. Decide about content or direction of a policy or program | 144 | 4 | 0.7 | 4 | 4 | 4 | 5 | 5 |  |
|  | - 1. Persuade others to a point of view or course of action | 144 | 4 | 0.7 | 3 | 4 | 4 | 5 | 5 |  |
|  | - 1. Design the implementation or evaluation strategy for a policy or program | 144 | 4 | 0.8 | 3 | 4 | 4 | 5 | 5 |  |
|  | - 1. Monitor implementation or evaluate the impact of a policy or program | 144 | 4 | 0.9 | 3 | 3 | 4 | 5 | 5 |  |
|  | - 1. Meet organisational requirements to use research | 143 | 4 | 1 | 2 | 3 | 4 | 5 | 5 |  |
| 1. **Confidence** individual has in their own knowledge and skills | I feel confident that I have the knowledge and skills to: |  |  |  |  |  |  |  |  |  |
|  | - 1. Find research to inform policy or program development | 144 | 4 | 0.9 | 3 | 3 | 4 | 5 | 5 | 5 point adjectival scale; not at all confident (1) to very confident (5) |
|  | - 1. Evaluate the quality of research | 144 | 4 | 1.1 | 2 | 3 | 4 | 4 | 5 |  |
|  | - 1. Interpret the results of research | 144 | 4 | 1 | 3 | 3 | 4 | 4 | 5 |  |
|  | - 1. Apply research to policy or program development | 144 | 4 | 0.9 | 3 | 3 | 4 | 5 | 5 |  |
|  | - 1. Design evaluations of policies or programs | 144 | 3 | 1 | 2 | 3 | 3 | 4 | 5 |  |
|  | - 1. Commission research to support policy or program development | 144 | 3 | 1.1 | 2 | 3 | 4 | 4 | 5 |  |
|  | - 1. Partner with researchers to generate research | 144 | 3 | 1.1 | 2 | 3 | 4 | 4 | 5 |  |
| 1. **Value organisation** places on using research | In my organisation: |  |  |  |  |  |  |  |  |  |
|  | - 1. Leaders believe it is important to use research in policy or program development | 144 | 4 | 0.8 | 3 | 4 | 4 | 5 | 5 | 5 point adjectival scale; never (1) to always (5) |
|  | - 1. It is expected that research will be used in policy or program development | 144 | 4 | 0.9 | 3 | 4 | 4 | 5 | 5 |  |
|  | - 1. Generation of new research to inform policy or program development is encouraged | 144 | 4 | 0.9 | 2 | 3 | 4 | 4 | 5 |  |
|  | - 1. It is expected that policies/programs will be evaluated | 144 | 4 | 1 | 2 | 3 | 4 | 5 | 5 |  |
|  | - 1. Interaction or collaboration with researchers or research organisations is encouraged | 144 | 4 | 0.9 | 3 | 3 | 4 | 4 | 5 |  |
| 1. **Tools and systems** organisation has to support research engagement actions and use | My organisation: |  |  |  |  |  |  |  |  |  |
|  | - 1. Has processes for policy or program development that provide guidance on how research should be used | 144 | 2 | 0.7 | 1 | 1 | 2 | 2 | 3 | 4 options; no (1), yes but limited (2), yes well developed (3), I don’t know (1) |
|  | - 1. Has systems that encourage leaders to support use of research | 144 | 2 | 0.7 | 1 | 1 | 2 | 2 | 3 |  |
|  | - 1. Provides access to training in using research in policy or program development | 144 | 2 | 0.7 | 1 | 1 | 2 | 2 | 3 |  |
|  | - 1. Has the resources needed to access research (e.g. subscriptions to journals, a library, relevant software) | 144 | 2 | 0.8 | 1 | 2 | 2 | 3 | 3 |  |
|  | - 1. Has established methods for commissioning reviews of research | 144 | 2 | 0.9 | 1 | 1 | 2 | 3 | 3 |  |
|  | - 1. Has documented processes for how policies or programs should be evaluated | 144 | 2 | 0.7 | 1 | 1 | 2 | 2 | 3 |  |
|  | - 1. Has existing relationships, or established methods for engaging, with research organisations | 144 | 2 | 0.7 | 1 | 2 | 3 | 3 | 3 |  |

* Response options are stated in words (e.g. “very confident”) when administering SEER without a numeric value.

Table S3. Item descriptive statistics for SEER research engagement scales

| Factors | Items | Response | n | Mean or % | SD | Distribution (percentile) | | | | | Response options (score)* |
| --- | --- | --- | --- | --- | --- | --- | --- | --- | --- | --- | --- |
|  |  |  |  |  |  | p10 | p25 | p50 | p75 | p90 |  |
| 1. **Accessed synthesised** research | For this area of policy or program work did you (or a member of staff at your direction): |  |  |  |  |  |  |  |  |  |  |
|  | - 1. Search for reviews of research summarising and evaluating the results of multiple studies (i.e. systematic reviews, meta-analyses)? | No | 39 | 27% |  |  |  |  |  |  | Binary response; no (0), yes (1) |
|  |  | Yes | 103 | 73% |  |  |  |  |  |  |  |
|  | - 1. Commission a review of research to summarise and evaluate the results of available studies? | No | 78 | 55% |  |  |  |  |  |  |  |
|  |  | Yes | 64 | 45% |  |  |  |  |  |  |  |
| 1. **Accessed primary** research | For this area of policy or program work did you (or a member of staff at your direction): |  |  |  |  |  |  |  |  |  |  |
|  | - 1. Search for research papers reporting the results of single studies (e.g. randomised controlled trials, qualitative studies)? | No | 36 | 25% |  |  |  |  |  |  | Binary response no (0), yes (1) |
|  |  | Yes | 106 | 75% |  |  |  |  |  |  |  |
|  | - 1. Search for research on government websites (e.g. AIHW)? | No | 31 | 22% |  |  |  |  |  |  |  |
|  |  | Yes | 111 | 78% |  |  |  |  |  |  |  |
| 1. **Appraised** research | For this area of policy or program work did you (or a member of staff at your direction) assess the usefulness of the research or review based on: |  |  |  |  |  |  |  |  |  |  |
|  | - 1. The appropriateness of methods used to answer the question | No | 25 | 20% |  |  |  |  |  |  | Binary response no (0), yes (1) |
|  |  | Yes | 97 | 80% |  |  |  |  |  |  |  |
|  | - 1. The likelihood that the methods used meant that the results were reliable (unbiased) | No | 22 | 18% |  |  |  |  |  |  |  |
|  |  | Yes | 100 | 82% |  |  |  |  |  |  |  |
|  | - 1. Generalisability of the findings to your context, based on similarity of the included population, health system or other factors | No | 13 | 11% |  |  |  |  |  |  |  |
|  |  | Yes | 108 | 89% |  |  |  |  |  |  |  |
| 1. **Generated** research | For this area of policy or program work did you (or a member of staff at your direction): |  |  |  |  |  |  |  |  |  |  |
|  | - 1. Undertake or participate in an internally conducted research project or analysis of data | No | 65 | 46% |  |  |  |  |  |  | Binary response no (0), yes (1) |
|  |  | Yes | 75 | 54% |  |  |  |  |  |  |  |
|  | - 1. Commission or partner with researchers to conduct a research project or analysis of data | No | 77 | 55% |  |  |  |  |  |  |  |
|  |  | Yes | 63 | 45% |  |  |  |  |  |  |  |
|  | - 1. Plan or undertake an evaluation of the program or policy | No | 70 | 50% |  |  |  |  |  |  |  |
|  |  | Yes | 70 | 50% |  |  |  |  |  |  |  |
| 1. **Interacted** with researchers | In the last six months I have: |  |  |  |  |  |  |  |  |  |  |
|  | - 1. Worked with researchers to identify policy or program direction/priorities, or research direction/priorities |  | 140 | 2 | 1.3 | 1 | 1 | 2 | 4 | 4 | 4 point adjectival scale; not at all (1), once (2), twice (3), more than twice (4) |
|  | - 1. Collaborated with researchers to develop or implement a research project |  | 140 | 2 | 1.2 | 1 | 1 | 2 | 3 | 4 |  |
|  | - 1. Collaborated on a competitive research grant application (e.g. NHMRC, ARC) |  | 140 | 1 | 0.7 | 1 | 1 | 1 | 1 | 2 |  |
|  | - 1. Contributed to analysis and/or writing up of research results, or to other aspects of a research publication |  | 140 | 2 | 1.1 | 1 | 1 | 1 | 3 | 4 |  |
|  | - 1. Attended forums (e.g. conferences, workshops, symposia) to hear about research findings |  | 140 | 2 | 1.1 | 1 | 2 | 2 | 4 | 4 |  |
|  | - 1. Acted in an advisory capacity to a research team (e.g. on a steering committee) |  | 140 | 2 | 1.2 | 1 | 1 | 1 | 3 | 4 |  |

* Response options are stated in words (e.g. “yes”, “no”, “once”) when administering SEER without a numeric value.

Table S4. Item descriptive statistics for SEER research use scales

| Factors | Items | Response | n | Mean or % | SD | Distribution (percentile) | | | | | Response options (score)* |
| --- | --- | --- | --- | --- | --- | --- | --- | --- | --- | --- | --- |
|  |  |  |  |  |  | p10 | p25 | p50 | p75 | p90 |  |
| 1. **Extent** of research use | In each stage of these policy or program development processes, to what extent was research used? |  |  |  |  |  |  |  |  |  |  |
|  | - 1. Agenda setting/scoping |  | 140 | 4 | 1.7 | 1 | 3 | 4 | 5 | 6 | 6 point scale; none (1), minimal (2), limited (3), moderate (4), high (5), extensive (6), N/A (0) |
|  | - 1. Policy or program development |  | 140 | 5 | 1.4 | 3 | 4 | 5 | 5 | 6 |  |
|  | - 1. Policy or program implementation |  | 140 | 3 | 2 | 0 | 1 | 4 | 5 | 5 |  |
|  | - 1. Policy or program evaluation |  | 140 | 2 | 2.1 | 0 | 0 | 3 | 4 | 5 |  |
| Type of research use | For this area of policy or program work did you (or a member of staff at your direction) use research: |  |  |  |  |  |  |  |  |  |  |
| 1. **Conceptual** research use | To help you understand how to think about an issue | No | 15 | 11% |  |  |  |  |  |  | Binary response no (0), yes (1) |
|  |  | Yes | 125 | 89% |  |  |  |  |  |  |  |
| 1. **Instrumental** research use | To decide about content or direction of a policy or program | No | 21 | 15% |  |  |  |  |  |  |  |
|  |  | Yes | 119 | 85% |  |  |  |  |  |  |  |
| 1. **Tactical** research use | To persuade others to a point of view or course of action | No | 23 | 16% |  |  |  |  |  |  |  |
|  |  | Yes | 117 | 84% |  |  |  |  |  |  |  |
| 1. **Imposed** research use | Because your organisation required you to use research | No | 74 | 53% |  |  |  |  |  |  |  |
|  |  | Yes | 66 | 47% |  |  |  |  |  |  |  |

* Response options are stated in words (e.g. “yes”, “limited”) when administering SEER without a numeric value.
