# Supplementary material for: Development and validation of SEER (Seeking, Engaging with and Evaluating Research): a measure of policymakers’ capacity to engage with and use research
Source: Health Res Policy Syst. 2017 Jan 17;15:1. doi: 10.1186/s12961-016-0162-8 (PMC5240393; doi:10.1186/s12961-016-0162-8)
Supplement: Additional file 3: — Theory of planned behaviour model and instrument. (DOCX 36 kb) [file 12961_2016_162_MOESM3_ESM.docx]

**Figure S1.** Theory of planned behaviour (TPB) constructs, relationships between them and how the constructs relate to the 12 item questionnaire (from Boyko et al Health Research Policy and Systems 2011, 9:29 http://www.health-policy-systems.com/content/9/1/29)

**A Tool for Measuring Theory of Planned Behaviour Constructs for use in**

**Evaluating Research Use in Policymaking^[[1]](#footnote-1)^**

**Behavioural intention**

1. I expect to use research evidence to help work through what I will say in policy documents or briefings, advocate for, or decide.

| Strongly disagree | Disagree | Somewhat  disagree | Neither agree nor disagree | Somewhat  agree | Agree | Strongly agree |
| --- | --- | --- | --- | --- | --- | --- |
| 1 | 2 | 3 | 4 | 5 | 6 | 7 |

1. I want to use research evidence to help work through what I will say in policy documents or briefings, advocate for, or decide.

| Strongly disagree | Disagree | Somewhat  disagree | Neither agree nor disagree | Somewhat  agree | Agree | Strongly agree |
| --- | --- | --- | --- | --- | --- | --- |
| 1 | 2 | 3 | 4 | 5 | 6 | 7 |

1. I intend to use research evidence to help work through what I will say in policy documents or briefings, advocate for, or decide.

| Strongly disagree | Disagree | Somewhat  disagree | Neither agree nor disagree | Somewhat  agree | Agree | Strongly agree |
| --- | --- | --- | --- | --- | --- | --- |
| 1 | 2 | 3 | 4 | 5 | 6 | 7 |

**Attitudes**

1. Using research evidence to help work through what I will say in policy documents or briefings, advocate for, or decide is…

| Very harmful | Moderately  harmful | Slightly harmful | Neutral | Slightly beneficial | Moderately beneficial | Very beneficial |
| --- | --- | --- | --- | --- | --- | --- |
| 1 | 2 | 3 | 4 | 5 | 6 | 7 |

| Very bad | Moderately bad | Slightly bad | Neutral | Slightly good | Moderately good | Very good |
| --- | --- | --- | --- | --- | --- | --- |
| 1 | 2 | 3 | 4 | 5 | 6 | 7 |

| Very unpleasant  (for me) | Moderately  unpleasant (for me) | Slightly  unpleasant  (for me) | Neutral | Slightly pleasant (for me) | Moderately pleasant (for me) | Very pleasant (for me) |
| --- | --- | --- | --- | --- | --- | --- |
| 1 | 2 | 3 | 4 | 5 | 6 | 7 |

| Very unhelpful | Moderately  unhelpful | Slightly unhelpful | Neutral | Slightly helpful | Moderately helpful | Very helpful |
| --- | --- | --- | --- | --- | --- | --- |
| 1 | 2 | 3 | 4 | 5 | 6 | 7 |

**Subjective norms**

1. Most people who are important to me in my professional life think that…

| I should definitely not | I should almost certainly not | I should probably not | Neutral | I should probably | I should almost certainly | I should definitely |
| --- | --- | --- | --- | --- | --- | --- |
| 1 | 2 | 3 | 4 | 5 | 6 | 7 |

… use research evidence to help work through what I will say in policy documents or briefings, advocate for, or decide.

1. It is expected of me that I use research evidence to help work through what I will say in policy documents or briefings, advocate for, or decide.

| Strongly disagree | Disagree | Somewhat  disagree | Neither agree nor disagree | Somewhat  agree | Agree | Strongly agree |
| --- | --- | --- | --- | --- | --- | --- |
| 1 | 2 | 3 | 4 | 5 | 6 | 7 |

1. I feel under social pressure to use research evidence to help work through what I will say in policy documents or briefings, advocate for, or decide.

| Strongly disagree | Disagree | Somewhat  disagree | Neither agree nor disagree | Somewhat  agree | Agree | Strongly agree |
| --- | --- | --- | --- | --- | --- | --- |
| 1 | 2 | 3 | 4 | 5 | 6 | 7 |

1. People who are important to me in my professional life want me to use research evidence to help work through what I will say in policy documents or briefings, advocate for, or decide.

| Strongly disagree | Disagree | Somewhat  disagree | Neither agree nor disagree | Somewhat  agree | Agree | Strongly agree |
| --- | --- | --- | --- | --- | --- | --- |
| 1 | 2 | 3 | 4 | 5 | 6 | 7 |

**Perceived behavioural control^[[2]](#footnote-2)^**

1. I am confident that I could use research evidence to help work through what I will say in policy documents or briefings, advocate for, or decide.

| Strongly disagree | Disagree | Somewhat  disagree | Neither agree nor disagree | Somewhat  agree | Agree | Strongly agree |
| --- | --- | --- | --- | --- | --- | --- |
| 1 | 2 | 3 | 4 | 5 | 6 | 7 |

1. For me to use research evidence to help work through what I will say in policy documents or briefings, advocate for, or decide is…

| Very difficult | Moderately difficult | Slightly difficult | Neutral | Slightly easy | Moderately easy | Very easy |
| --- | --- | --- | --- | --- | --- | --- |
| 1 | 2 | 3 | 4 | 5 | 6 | 7 |

1. The decision to use research evidence to help work through what I will say in policy documents or briefings, advocate for, or decide is beyond my control.

| Strongly disagree | Disagree | Somewhat  disagree | Neither agree nor disagree | Somewhat  agree | Agree | Strongly agree |
| --- | --- | --- | --- | --- | --- | --- |
| 1 | 2 | 3 | 4 | 5 | 6 | 7 |

1. Whether or not I use research evidence to help work through what I will say in policy documents or briefings, advocate for, or decide is entirely up to me.

| Strongly disagree | Disagree | Somewhat  disagree | Neither agree nor disagree | Somewhat  agree | Agree | Strongly agree |
| --- | --- | --- | --- | --- | --- | --- |
| 1 | 2 | 3 | 4 | 5 | 6 | 7 |

1. Instrument modified from: Boyko J, Lavis J, Dobbins M, et al. Reliability of a Tool for Measuring Theory of Planned Behaviour Constructs for use in Evaluating Research Use in Policymaking. Health Research Policy and Systems 2011;9(1):29 [↑](#footnote-ref-1)
2. Items 9 and 10 measure self-efficacy. [↑](#footnote-ref-2)
