# Supplementary figures and images for: Development and validation of SEER (Seeking, Engaging with and Evaluating Research): a measure of policymakers’ capacity to engage with and use research
Source: Health Res Policy Syst. 2017 Jan 17;15:1. doi: 10.1186/s12961-016-0162-8 (PMC5240393; doi:10.1186/s12961-016-0162-8)

# Empirical distributions of responses to SEER and TPB scales

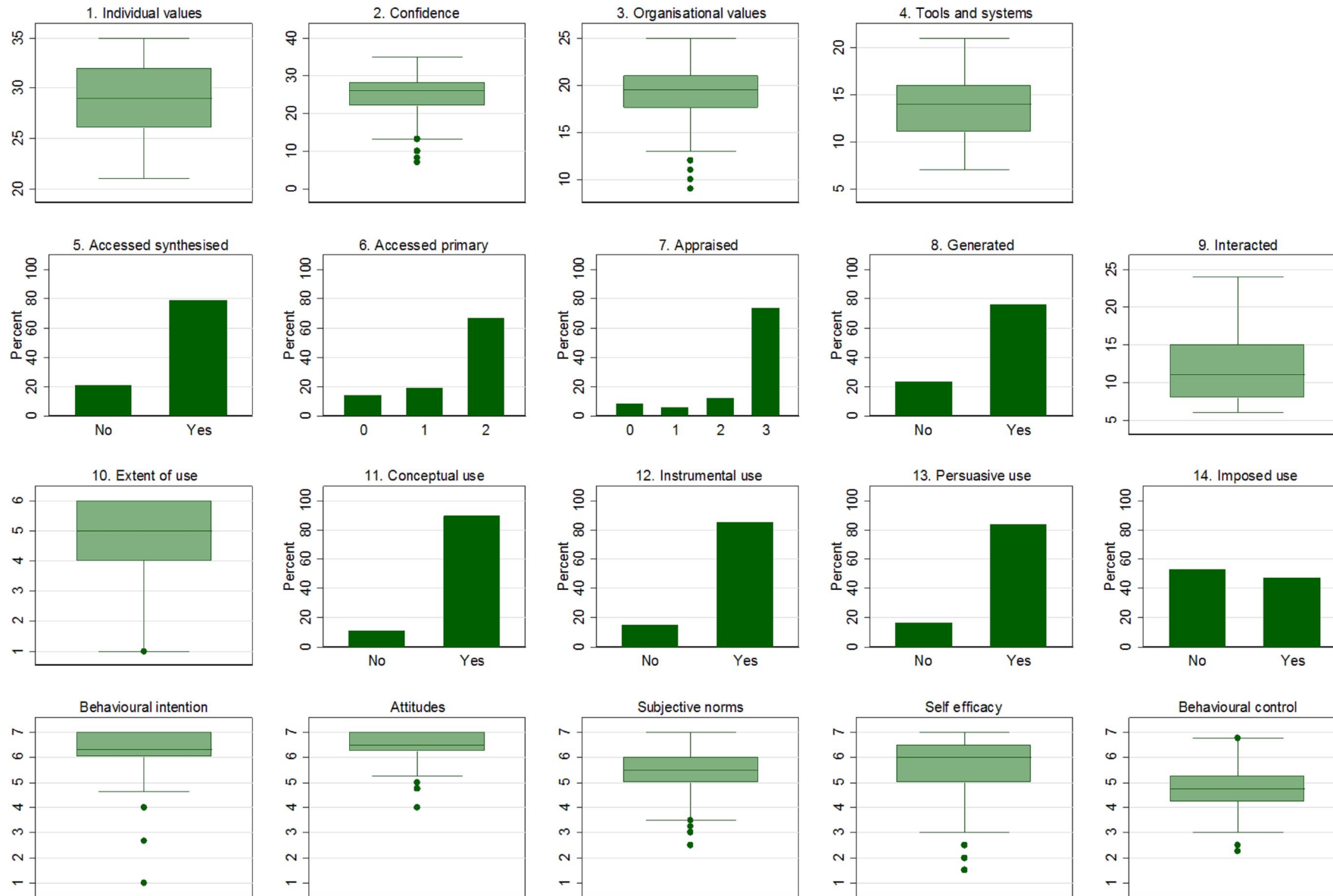

Supplement: Additional file 4: — Plots showing distribution of SEER scale scores. (PDF 204 kb) [file 12961_2016_162_MOESM4_ESM.pdf]
