# Supplementary material for: Development and validation of SEER (Seeking, Engaging with and Evaluating Research): a measure of policymakers’ capacity to engage with and use research
Source: Health Res Policy Syst. 2017 Jan 17;15:1. doi: 10.1186/s12961-016-0162-8 (PMC5240393; doi:10.1186/s12961-016-0162-8)

Plots of the association between SEER capacity scales and TPB measures

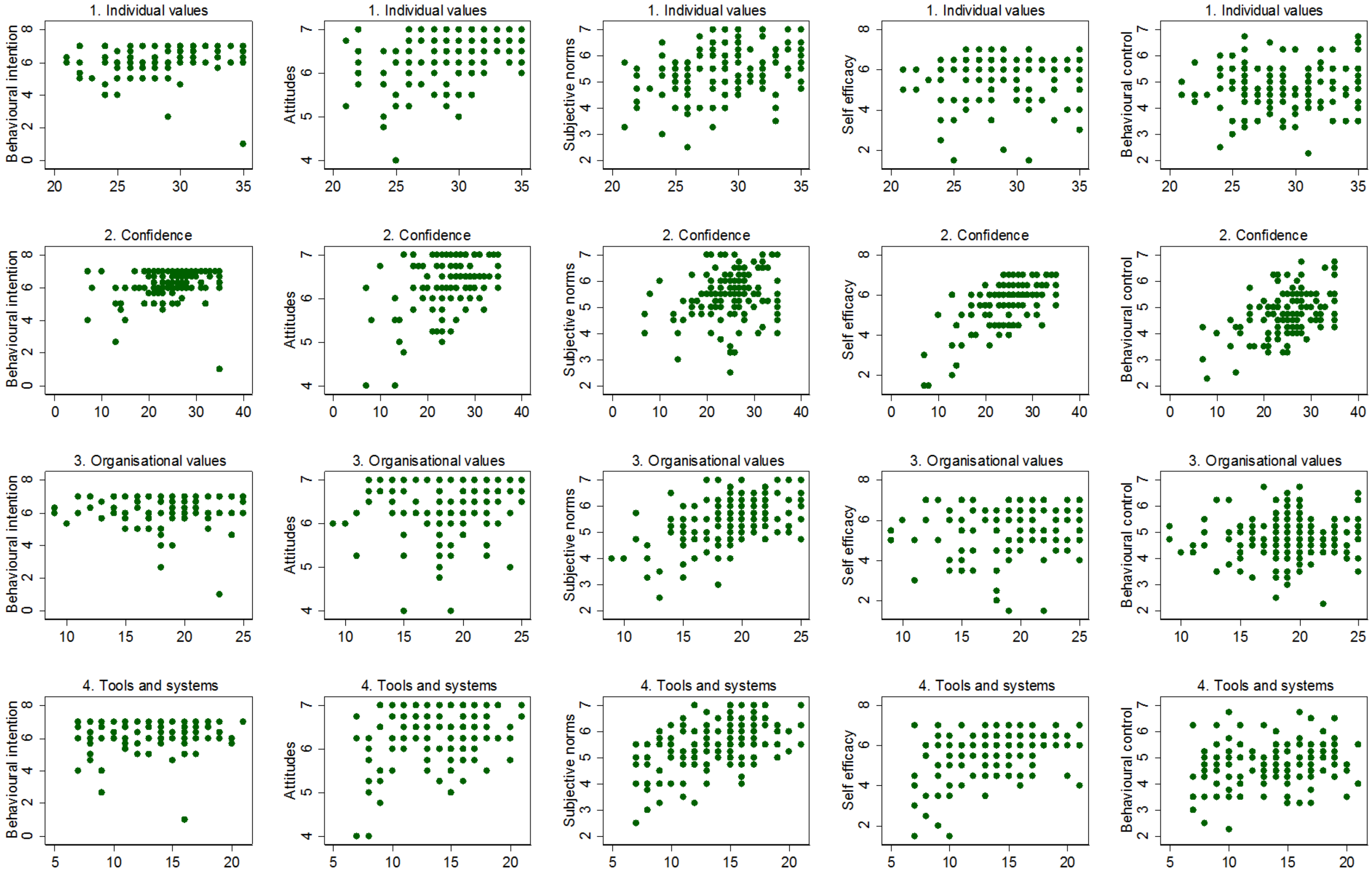

Plots of the association between SEER research engagement scales and TPB measures

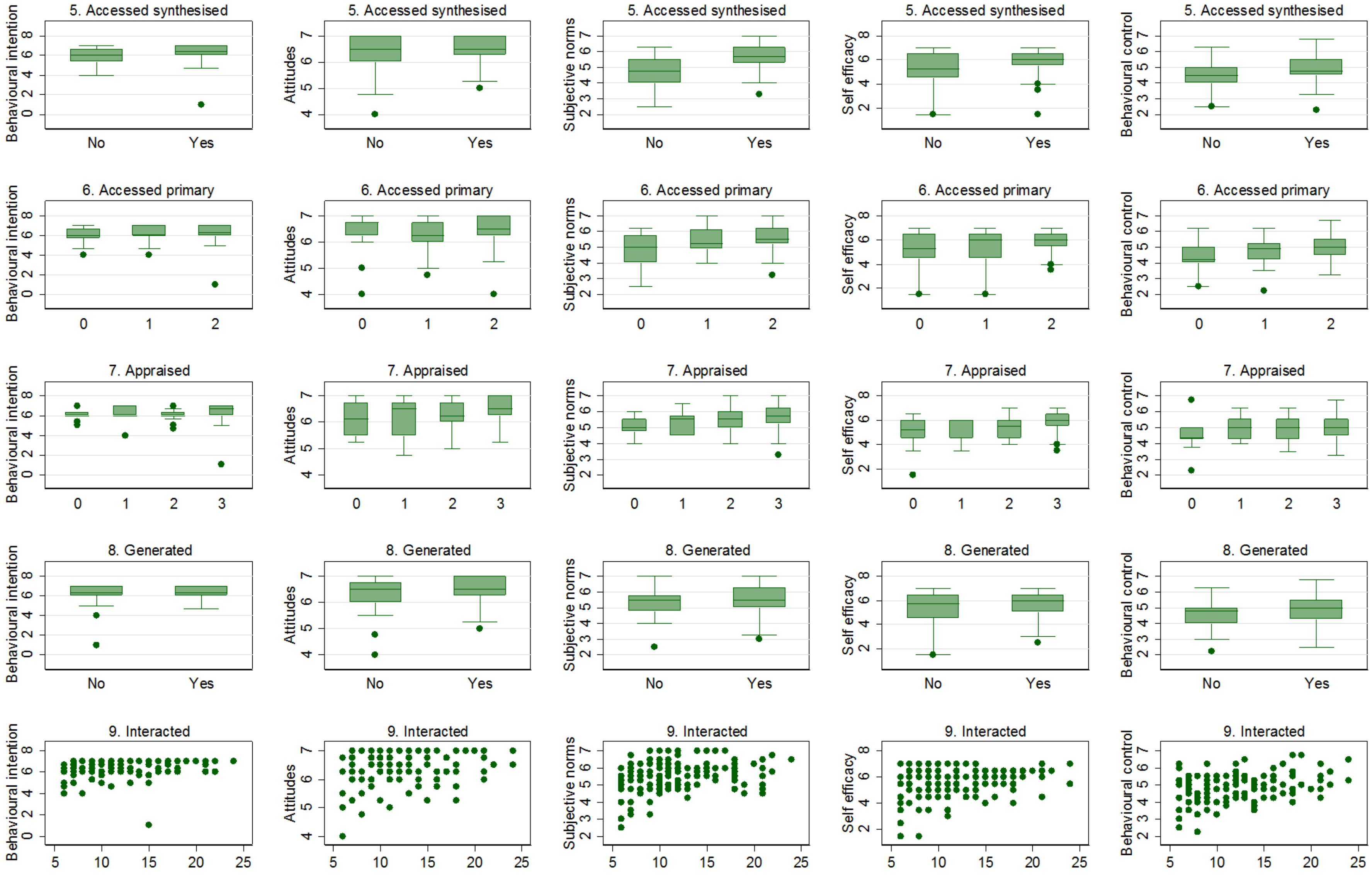

Plots of the association between SEER research use scales and TPB measures

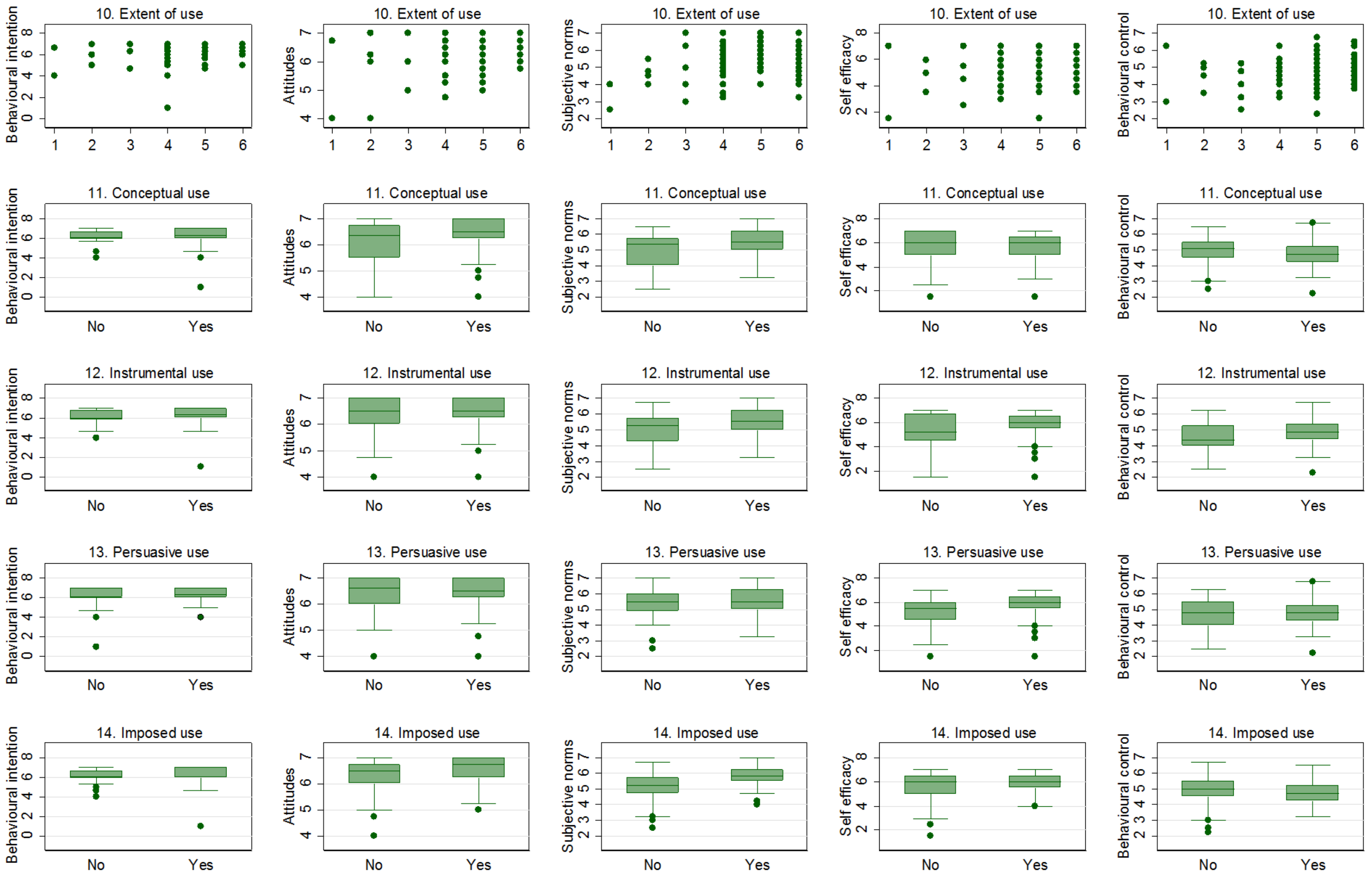

Supplement: Additional file 6: — Plots showing association between SEER and theory of planned behaviour scale scores. (PDF 189 kb) [file 12961_2016_162_MOESM6_ESM.pdf]
